# Sexual Orientation Disclosure and Strategic Navigation of Interpersonal Invisibility

**DOI:** 10.1177/01461672241313269

**Published:** 2025-01-24

**Authors:** Emily Schwartzman, Rebecca Neel

**Affiliations:** 1 University of Toronto, ON, Canada

**Keywords:** interpersonal invisibility, sexual orientation, stigma, identity management

## Abstract

People with concealable stigmatized identities may strategically share or hide cues to their identity. They may likewise seek or avoid interpersonal invisibility (i.e., being ignored). Despite semantic similarities, identity concealment and invisibility are conceptually distinct, but existing empirical work has not explored whether they are independent—that is, whether people may sometimes reveal to be invisible, or conceal to be visible. To evaluate this distinction, we collected lesbian, gay, and bisexual (LGB) participants’ free-response descriptions of situations where they revealed or concealed their sexual orientation to be visible or invisible (Study 1). Other LGB participants imagined social interactions in which they revealed or concealed their sexual orientation to be visible or invisible and rated their expected emotions in this situation (Study 2). We find that concealment and invisibility can occur independently of one another with separate effects on emotions, and that LGB people use identity disclosure and concealment to strategically navigate interpersonal invisibility.

A person who has a concealable stigmatized identity faces the ongoing question of whether and when to reveal or conceal their potentially stigmatizing identity. People use concealment and disclosure to manage their interactions with others, seeking to manage potential prejudice and discrimination (Goffman, 1963), for example, or to build relationships with sympathetic others (Chaudoir & Fisher, 2010). In the current research, we examine whether people also use concealment and disclosure to manage *interpersonal invisibility*, a form of stigmatization in which one is ignored and overlooked by others.

At first blush, interpersonal invisibility may seem similar to concealment: Both involve something or someone being unseen, unknown, or unacknowledged. One might assume that concealment is primarily used to seek invisibility and avoid unwanted attention, such as prejudiced behavior. However, based on reasoning from the affordance management theory of interpersonal invisibility (Neel & Lassetter, 2019), we anticipate that concealment can also be a strategy that people use to seek *visibility*. That is, people may sometimes conceal their potentially stigmatizing identity so that others will see them as relevant and worthy of attention. Likewise, people may sometimes disclose their identity to avoid unwanted attention (i.e., to deliberately seek invisibility).

The present research examines whether desires for interpersonal visibility and invisibility shape decisions to reveal or conceal a stigmatized identity. Using qualitative and quantitative methods, we examine lesbians, gay men, and bisexuals’ decisions to conceal or reveal their sexual orientation, and the motivations that accompany these decisions. Specifically, we examine whether they at times use each of these strategies to seek invisibility *and* visibility: Do they sometimes reveal their sexual orientation to be invisible to others, and sometimes conceal their sexual orientation to be visible to others? We furthermore explore the emotional effects of these decisions to understand how the affective experience of seeking invisibility may differ from the affective experience of concealing one’s identity.

## Invisibility by Perceived Irrelevance

The affordance-management theory of interpersonal invisibility (Neel & Lassetter, 2019) provides a framework for understanding when people will experience different forms of stigmatization across different situations. The theory posits that stigmatization reflects devaluation (Crocker et al., 1998; Goffman, 1963; Jones, 1984) and comes in two general forms: threat-based stigmatization and interpersonal invisibility. Whereas threat-based stigmatization manifests as negative prejudices, attention, and discriminatory behaviors, interpersonal invisibility manifests as indifference and inattention.

A person who is devalued will be subjected to threat-based stigmatization or to interpersonal invisibility depending on the affordances (i.e., opportunities and/or threats) others believe they present: People experience threat-based stigmatization when they are perceived to threaten others’ goals, whereas they experience interpersonal invisibility when others judge them to be goal-irrelevant. For ease, we call the person who may be stigmatized the “target” and the person who may stigmatize the target the “perceiver.” Perceivers may use various forms of information about the target, including the targets’ identity, as affordance cues that signal what threats and opportunities the target may present. For example, because of stereotypes that Asian students are academically strong, a White student perceiver who is competing for an academic award might judge an Asian classmate to be a goal threat (Zhang, 2010). Based on this perception, the White student may engage in threat-based stigmatization of their Asian classmate, paying more attention to them (e.g., monitoring the classmate’s grades), feeling negative emotion toward them (e.g., envy, anger), and engaging in discriminatory behaviors (e.g., refusing to share notes). In contrast, another White student perceiver competing for an athletic award might judge that same Asian classmate to be entirely irrelevant to their goal, due to the stereotype that Asian people lack physical or athletic ability (Wong et al., 2013). This second perceiver would then subject the target to interpersonal invisibility, ignoring and neglecting them altogether. Thus, stigmatized targets may experience hostility and negative attention from those who see them as a threat, and inattention and neglect from those who see them as goal-irrelevant.

Of course, targets have their own goals, too. Targets may find that being perceived as threatening or irrelevant to others may sometimes pose an obstacle to their own aims, such as seeking safety and belonging. For example, if the Asian student wants to be a competitive athlete, being invisible to others may hurt that goal, and so they may seek to advertise their strength to be seen as a viable competitor or valued teammate. Or, to avoid the hassle and demeaning implications of being stereotyped as an academic threat, the Asian student might seek to be invisible to the other student. Thus, in service of their own goals, targets may seek to actively shape whether others perceive them to be relevant or irrelevant.

Targets will, moreover, have their own emotional responses to perceivers’ appraisals of their relevance. The affordance-management theory of interpersonal invisibility directly addresses perceivers’ emotional experiences—namely, experiencing negative emotion toward targets appraised as threatening and experiencing no emotion toward targets appraised as irrelevant—but is less clear about implications for targets’ emotional experiences. Thus, the emotions associated with targets’ experiences of invisibility merit exploration alongside targets’ goals.

Note that in these examples, the perceivers used the Asian student’s race as a cue to academic and athletic ability. To determine relevance to any goal, perceivers integrate a variety of affordance cues, such as group membership, facial expression, and many others, relying on whichever cues are available and considered informative (Kawakami et al., 2017; Neel & Lassetter, 2019). Importantly, this means that targets can manage others’ judgments by displaying or hiding particular affordance cues (e.g., a person at a singles event might smile so that others see them as approachable). Many identities that are considered meaningful affordance cues, such as race, are often hard to conceal. However, targets who feel their identities are concealable can elect to conceal or reveal that identity to perceivers (Le Forestier et al., 2022). We expect that targets will selectively reveal or conceal their stigmatized identity, depending on how they wish to be evaluated by others. Thus, the affordance-management perspective suggests the conceptual distinction between concealment and invisibility: Whereas invisibility is about *being perceived as irrelevant based on available cues*, concealment is about *managing which cues about you are available to others* and thus, the ways that you will be seen as relevant or irrelevant to others’ goals.

Under this framework, visibility and invisibility arise from another person’s appraisal of a target’s relevance to that person’s goals, whereas disclosure and concealment arise from the target’s behavior. However, disclosure and concealment can directly influence a target’s visibility depending on the other person’s active goal and whether the target’s concealable identity is seen as signaling relevance or irrelevance to that goal. If a given concealable identity is viewed as a cue that someone is irrelevant to a particular goal, then a target who holds that identity will be visible if they conceal their identity or invisible if they disclose their identity. However, if a given concealable identity is viewed as a relevance cue for a particular goal, then a target will be invisible if they conceal their identity or visible if they disclose their identity. Thus, if a target believes their identity will serve as a cue to their relevance or their irrelevance to the other person’s goals, it is possible that they might strategically elect to reveal or conceal their identity to deliberately attain visibility or invisibility depending on their own aims.

**Figure 1:**
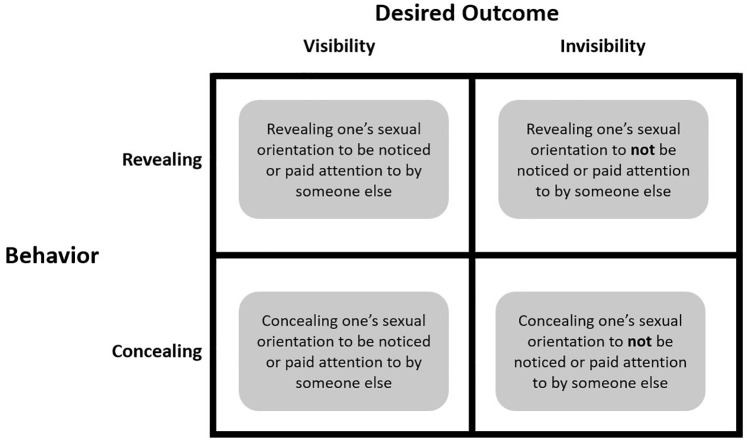
Theoretical Framework Separating Concealment From Invisibility.

Consider sexual orientation. A sexual minority person might disclose their sexual orientation to become visible (e.g., a gay man outing himself to another gay man to be seen as a viable romantic partner) or invisible (e.g., a lesbian outing herself to a straight man to avoid unwanted romantic attention). Likewise, they may conceal their sexual orientation to render themselves visible (e.g., to avoid being devalued as an employee in the workplace) or invisible (e.g., to avoid being targeted by homophobic violence; as hypothesized in Neel & Lassetter, 2019). This theoretical distinction between concealment and invisibility has not yet been empirically evaluated. We focus specifically on disclosures of sexual orientation as an initial empirical test of this conceptual distinction (Figure 1).

## Sexual Orientation and Outness

A large body of research examines “coming out” decisions among those with sexual minority identities, making this a well-justified context for examining how interpersonal invisibility relates to concealment. Rather than being a one-time event, coming out is an ongoing process (Orne, 2011). The decision to reveal or conceal one’s sexual orientation must be made repeatedly, and the same individual may make different decisions in different contexts: An individual who is “out” to their friends and family may still choose to conceal their sexual orientation in particular situations (Malterud & Bjorkman, 2016). Thus, sexual minority individuals must repeatedly decide whether or not to share information about their identity.

Recent literature distinguishes active concealment (deliberate actions taken to avoid revealing one’s sexual orientation, such as lying or avoiding interactions with others) from nondisclosure (passively refraining from sharing one’s sexual orientation without the use of additional concealment strategies), with active concealment more strongly predicting relevant outcomes such as well-being (Camacho et al., 2020; Quinn et al., 2017). Nevertheless, both of these behaviors reflect LGB people’s continual regulation of the information they make available about their sexual orientation, with this regulation leading to potential consequences for their well-being and emotional experiences. Indeed, previous work has identified that concealment is emotionally costly: Compared with those who disclose their sexual orientation in a given context, those who conceal tend to report more negative emotions and worse emotional well-being (e.g., Dhanani et al., 2024; Quinn et al., 2017). That said, little is known about the emotional consequences of experiencing interpersonal invisibility. Whereas social invisibility and the associated experience of being ignored and overlooked may be expected to elicit negative emotion (Leary, 1990; O’Reilly et al., 2015), it is also plausible that a target who deliberately seeks to become invisible would be less adversely affected.

Overall, past research has largely focused on how disclosure may be shaped by anticipated discrimination, prejudice, or a lack of support. Here, we focus on when people specifically choose to disclose or reveal their identity to manage perceptions of irrelevance and interpersonal invisibility and how these decisions relate to various goals (including not only the avoidance of stigma, but also the advancement of other social motivations). We furthermore explore the emotional effects of identity disclosure and concealment, and of social visibility and invisibility, to better understand the emotional consequences of both disclosure decisions and targets’ experiences of invisibility.

## The Current Research

We use mixed methods to examine whether participants with a concealable stigmatized identity distinguish between concealment and invisibility, and whether disclosure decisions depend on how invisibility would fit with participants’ goals. We also examine how participants’ goals may differ across the quadrants of our theoretical framework (Figure 1). Across the measures and manipulations used in these studies, we operationalize invisibility in terms of attention (e.g., “How much attention did the other person pay you?”). This is because the meaning of “invisibility” in a social context may be unclear to research participants or differ from our conceptualization of it, whereas paying someone more or less attention is a recognizable concept that also characterizes a target’s subjective experiences of social visibility and invisibility (Neel & Lassetter, 2019).

In Study 1, sexual minority research participants described past experiences where they either revealed or concealed their sexual orientation to become either visible or invisible to another person. In Study 2, a new sample of participants imagined hypothetical situations in which they revealed or concealed their sexual orientation to be either visible or invisible and reported the emotions they expected to experience in this situation. We report all measures, manipulations, and exclusions below. In a supplemental study, we used vignettes adapted from Study 1 participant responses to examine a new sample of participants’ predictions about a fictional character’s disclosure decisions. We report all measures, manipulations, and exclusions for this study in the Online Supplement.

## Study 1

We began by examining whether LGB individuals meaningfully distinguish between identity concealment and social invisibility in their own lived experiences. We took a qualitative research approach in which we prompted participants to write about a past experience that fits one of the four quadrants of this framework (i.e., either revealing or concealing their sexual orientation to seek either visibility or invisibility) and coded whether their responses fit the relevant behavior (revealing or concealing) and outcome (visibility or invisibility). This allowed us to examine whether participants report experiences that are consistent with this distinction: If participants can recall specific experiences in which they revealed their sexual orientation to become invisible or concealed their sexual orientation to become visible, this would demonstrate that concealment is not interchangeable with invisibility.

In addition to probing for descriptions of the experiences themselves, we also asked participants to describe their personal feelings and goals in these situations, and their reasons for either disclosing or not disclosing. Thus, beyond empirically testing the distinction between concealment and invisibility, we also examined the motives associated with these disclosure decisions, and how they vary across the different categories of responses participants provided.

### Method

Experimental materials and data for this study and for Study 2 are available on OSF (https://osf.io/h5p4n/?view_only=e3e3ada2911d4ce39c5cee3bd1e11cac). Study 1 was not preregistered.

#### Participants

Determining *a priori* sample size for qualitative research is a complicated methodological question with no clear best practices (see Sim et al., 2018). We therefore aimed to recruit a sample of approximately 200 participants on the basis that this would comfortably exceed the largest rule-of-thumb guidelines (which recommend up to 60 participants) while remaining manageable for coding purposes. We recruited a sample of 205 lesbian, gay, and bisexual participants residing in the United States or Canada using Prolific. After excluding 21 participants who met the study’s screening criteria on Prolific but identified themselves as “Straight/Heterosexual” in the survey, we obtained a final sample of 184 participants (*M*_age_ = 29.00, *SD* = 8.56). This sample comprised 129 women (including four transgender women, three nonbinary women, one genderfluid woman, and one agender woman), 42 men (including four transgender men), seven nonbinary participants, three participants of other genders, and three participants who did not indicate their gender. 112 participants reported their sexual orientation as bisexual, 51 as gay or lesbian, 11 as pansexual, one as asexual, five participants reported other sexual orientations, and four participants did not indicate their sexual orientation.

#### Procedure

##### Free-Response Task

After providing informed consent and their Prolific ID, participants were prompted to describe a previous experience. The first portion of the prompt probed for an instance where the participant either revealed their sexual orientation to someone else or concealed their sexual orientation from someone else. The second portion specified that the instance should be either a time when the participant “wanted the other person to notice or pay attention to [them]” (i.e., a time when they wished to be visible to the other person), or a time when they “didn’t want the other person to notice or pay attention to [them]” (i.e., a time when they wished to be invisible to the other person). The full text of the four possible prompts is available on OSF (https://osf.io/h5p4n/?view_only=e3e3ada2911d4ce39c5cee3bd1e11cac; see Study 1 Materials). This produced a two (behavior: reveal or conceal) by two (goal: visibility or invisibility) design, with participants randomly assigned to one of the four conditions.

Participants answered four free-response questions about their experience, which they were instructed to answer with as much detail as possible. These questions were:

1. “What exactly happened?”
2. “Who was involved?”
3. “What were you thinking, feeling, and wanting in this situation?”
4. “Why did you decide to share your sexual orientation?” (reveal condition) or “Why did you decide to avoid sharing your sexual orientation?” (conceal condition).

Following these questions, participants rated how easy it was to think of an experience that matched the prompt, using a 7-point Likert-type scale from 1 (*Not at all easy*) to 7 (*Very easy*). Our analytic approach and codebook for this qualitative data can be found in the Online Supplement.

##### Situation Ratings

Participants indicated whether or not the other person was aware of their sexual orientation, and how much attention the other person paid to them as a result, using 7-point Likert-type scales. For exploratory purposes, we also collected ratings of overall positive or negative emotions and of specific emotions, including shame, anxiety, relief, authenticity, and happiness (we report the results of these analyses in the Online Supplement). Given that many social interactions now take place virtually rather than in-person, we also included an exploratory item asking participants to indicate whether the situation they described took place in person or online/through other technology.

##### Other-Person Ratings

As an additional exploratory measure, we also asked participants to provide information about the person with whom they interacted in the situation they described, to the best of their ability. This information included basic demographics (gender, age, and race), beliefs/ideologies (religious and political), and various attributes of the dyadic relationship between the participant and the other person (the nature and closeness of their relationship, and relative power and social status). Because these measures were included for exploratory purposes, we do not report findings from them here.

Finally, participants provided demographic information about themselves and were debriefed.

### Results

#### Congruence Between Assigned Condition and Response

To explore how readily participants can generate experiences that reflect the quadrants of our theoretical framework, we began by comparing participants’ assigned prompts to the behavior and outcome they described in their response. Two trained research assistants coded all responses. We developed a deductive codebook prior to coding that provided criteria for classifying responses according to the described behavior (revealing or concealing) and outcome (visibility or invisibility) and for identifying motives present in the response. One of the authors met weekly with the coders throughout the coding process to iteratively expand and refine this codebook as needed (see the Online Supplement for a detailed overview of the coding process as well as the inclusion/exclusion criteria for the various codes). We note that our qualitative coding team ruled that the same response may receive both “reveal” and “conceal” behavior codes (e.g., a bisexual woman might present herself as solely attracted to women, simultaneously revealing her sexual minority status and concealing her true sexual orientation). Based on these codes, we then evaluated congruence between the prompt received and the response provided (Table 1). We conducted a chi-square test in R (Version 4.1.1; R Core Team, 2021) using the rstatix package (Kassambara, 2022) and the lsr package (Navarro, 2015). We found that the rate of congruence varied by assigned prompt, χ^2^(3) = 19.22, *p* < .001, Cramer’s *V* = .32: Post hoc analysis of residuals with Bonferroni-corrected *p* values showed that, compared with the overall rate of congruent answers pooled across prompt conditions, participants assigned to the reveal/visible prompt were more likely to provide responses congruent with the prompt (*p* = .005), whereas participants assigned to the reveal/invisible prompt were less likely to provide congruent responses (*p* = .002). Congruence rates for the conceal/visible and conceal/invisible prompts did not differ from the overall frequency of congruent responses (both *p*s > .999). Thus, different prompts show different likelihoods of eliciting suitable (i.e., congruent) responses from participants. Nevertheless, all four quadrants of our framework are represented within our qualitative data, suggesting that participants’ experiences can reflect each of the four quadrants. For all subsequent analyses, we retain these incongruent responses, but we group the data according to the responses that participants actually provided, as opposed to the conditions to which they were assigned (e.g., if a participant was prompted to describe concealing to become visible but our raters blindly determined that their response reflected concealing to become invisible, we analyzed their response alongside other responses about concealing to become invisible; Table 2).

**Table 1.** Congruent and Incongruent Responses by Assigned Condition.

Assigned condition	Congruent responses	Incongruent responses
Reveal, Visible	43	5
Reveal, Invisible	21	23
Conceal, Visible	31	13
Conceal, Invisible	34	14

**Table 2.** Total Responses by Coded Response Type.

Response type	Total responses
Reveal, Visible	58
Reveal, Invisible	24
Conceal, Visible	37
Conceal, Invisible	51
Multiple applicable response types	4
No applicable response type	10

*Note.* Response type corresponds to the content of the response as evaluated by qualitative coders, irrespective of the condition to which the participant was assigned.

Despite this significant variation in response congruence across conditions, we found no significant differences in participants’ self-reported ease of responding to their assigned prompt (see Online Supplement, Tables S1A & S1B for complete statistics).

#### Revealing to be Visible: Responses and Motives

**Figure 2.**
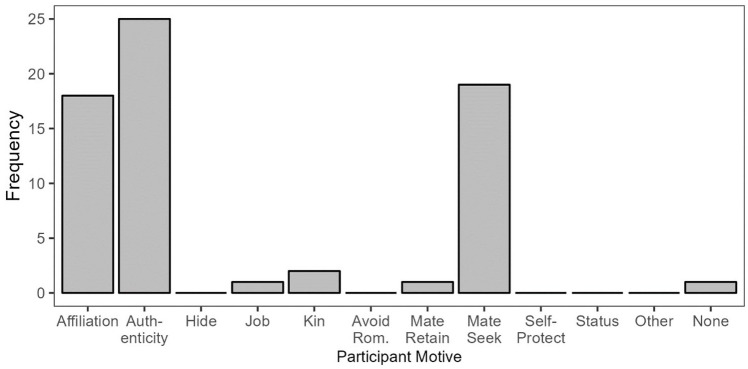
Participant Motives in Reveal/Visible Responses. *Note.* Because multiple motives could be assigned to the same participant, the total count of motives depicted in the figure exceeds the number of responses in this category.

Among the participants who described revealing their sexual orientation to be visible to others, three prevalent participant motives emerged: affiliation, authenticity, and mate-seeking (Figure 2).

Many participants who revealed their sexual orientation to become visible with the personal goal of affiliating described revealing to another person (typically a friend, coworker, or classmate) who they knew or suspected to be queer, believing that they would be accepted and that their shared experiences would strengthen their bond with the other person. Several participants likewise mentioned revealing their sexual orientation to signal belonging in LGBTQ+ spaces: For instance, a participant described mentioning to another person at an LGBTQ+ support center that they were in a relationship with a person of a different gender but then telling the other person that they are bisexual so that this person would not question the participant’s presence at the support center.

Participants who described revealing their sexual orientation to become visible and who were motivated by authenticity largely described interactions with friends, coworkers, or family members. These participants expressed wanting to belong and be accepted by others without the need for concealment and expressed trust in the other person. That said, several participants who were motivated by authenticity described revealing their sexual orientation to confront others’ prejudice or educate them (e.g., a gay man telling his coworkers about his long-term boyfriend to confront their stereotyping of gay men as promiscuous).

Finally, many participants who revealed their sexual orientation to become visible were motivated by mate-seeking. These participants largely revealed to strangers or friends, typically either to signal their eligibility as a romantic partner (in the case of strangers), or to “test the waters” of potentially initiating a relationship with a friend by gauging the friend’s reaction and seeing whether the friend would disclose a minority sexual identity in return.

#### Revealing to be Invisible: Responses and Motives

**Figure 3:**
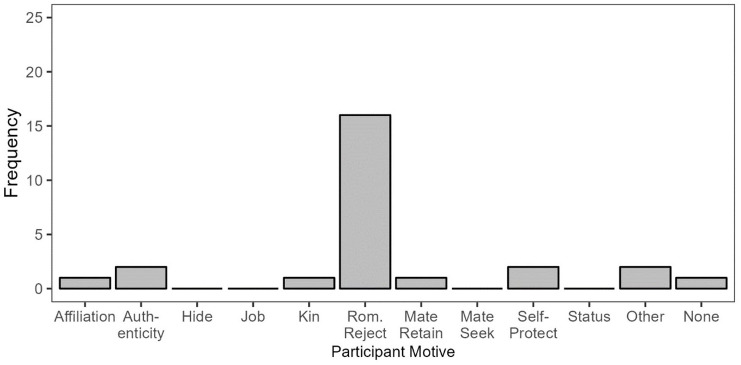
Participant Motives in Reveal/Invisible Responses.

Participants who described revealing their sexual orientation to become socially invisible displayed relatively uniform motives, with the motive to avoid unwanted romantic interest predominating (Figure 3). The vast majority of participants in this condition described disclosing their sexual orientation to someone (usually a coworker, classmate, or stranger) to dissuade romantic or sexual overtures. This occurred most straightforwardly in lesbian and gay participants, whereas for bisexual and pansexual participants, these disclosures involved some degree of simultaneous concealment where the participant downplayed their attraction to the gender of the person making unwanted advances. One participant acknowledged this directly, prefacing the specific situation they described by writing “I’m bisexual, but when faced with uncomfortable moments of pushy men, I’ve had moments of implying that I was mainly into girls.” Thus, people who experience attraction to multiple genders may respond to unwanted romantic interest by revealing their attraction to a different gender than that of the person expressing unwanted interest, and in doing so, simultaneously concealing their attraction to the gender of the person making unwanted advances.

#### Concealing to be Visible: Responses and Motives

**Figure 4:**
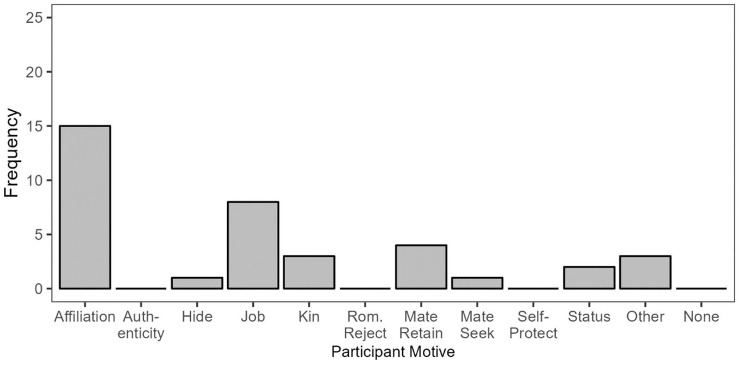
Participant Motives in Conceal/Visible Responses.

For participants who concealed their sexual orientation for the sake of visibility, affiliation was the most common motive, followed by job-related motives, and mate retention (Figure 4).

Recall that the affiliation motive was also common among participants who *revealed* for the sake of visibility. There are, however, some notable differences between how participants describe concealing for affiliation motives and revealing for affiliation motives. Participants who concealed for the sake of affiliation often noted that the other person was a straight man or highly religious, or else referenced a short acquaintanceship or the fact that the other person (typically a coworker) had previously expressed homophobic views. In contrast, participants in the reveal/visible response category who were motivated by affiliation often noted that they knew or suspected the other person to be queer or otherwise noted a close or trusting relationship. In a variation on this theme, the conceal/visible response category included one response from a bisexual woman who described referring to her boyfriend as her “partner” to avoid being mistaken for straight by a gay coworker, to be closer to him as friends. This was coded as concealment in that she concealed the gender of her partner, yet it otherwise resembles the situations described by participants who gave reveal/visible responses in that she signaled her sexual minority identity to strengthen her bond with a fellow sexual minority person.

We also saw several instances of the job motive among those who concealed to be visible. We had added this motive to the coding scheme in large part due to the numerous responses in which participants described concealing their sexual orientation in job interviews to be seen as a more viable candidate. Similarly, one participant described concealing their sexual orientation during a Pride event at work while they were up for promotion, out of concern that their identity would take attention away from the quality of their work.

The third most common motive in participants who concealed their sexual orientation to be visible was mate retention, though this only corresponded to four responses. These were all cases of bisexual participants who hid their sexual orientation from a straight partner, either in the early stages of their relationship or in one case, indefinitely, to avoid making their partner lose interest or feel insecure and thus remain visible to them as a viable mate.

#### Concealing to be Invisible: Responses and Motives

**Figure 5.**
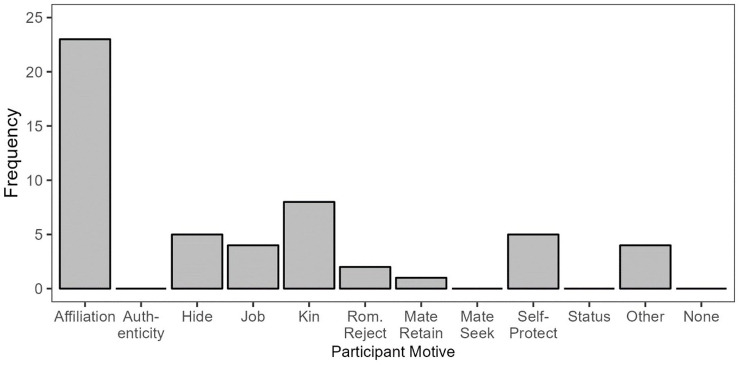
Participant Motives in Conceal/Invisible Responses.

Finally, for responses in which participants concealed their sexual orientation with the aim of invisibility, the most common motives were affiliation, kinship, hiding, and self-protection (Figure 5).

Again, we saw numerous cases of concealing from friends and coworkers for the purpose of affiliation. These responses were coded as invisible largely because the respondents explicitly mention not wanting to cause a scene, attract attention, or be subject to prejudice or stereotyping, thus avoiding unpleasant forms of visibility such as intrusive questions or homophobic harassment from their friends or colleagues. In contrast, participants concealing for visibility were those who described wanting to preserve a friendly relationship or allow a new friendship to develop, thus remaining relevant to the other person in the context of affiliation. In other words, responses coded as concealing for invisibility reflected a motive to avoid undesirable forms of attention, whereas responses coded as concealing for visibility reflected a motive to seek or maintain desirable forms of attention. Of note within these affiliation-motivated responses are two situations described by bisexual participants: One bisexual woman described concealing the gender of her male partner in LGBTQ+ social spaces, and another described concealing her bisexuality from a lesbian friend who was expressing negative views of bisexual people.

The next most common motive within this response type was kinship motive: This represents a somewhat broad interpretation of the fundamental kin care motive that also includes the drive to maintain good relationships with family members. Numerous participants described concealing their sexual orientation from family members to avoid possible or definite negative reactions. The inductively developed hide motive is closely related to these situations, as all five instances of the hide motive in this category occurred in interactions with family members. The hide motive was applied to responses that described feeling unready or uncomfortable with coming out in the absence of clear concerns about relationships or safety (e.g., “I wasn’t incredibly uncomfortable because I know my parents would accept me either way, but I just didn’t want to talk about it just then.”).

The last prominent motive in this category was self-protection. Responses corresponding to this motive described concealing from various others who participants perceived as posing some kind of danger, either because the other person was outwardly homophobic or because they held a position of power relative to the participant (e.g., police officers, doctors).

### Discussion

Study 1 used the affordance-management theory of interpersonal invisibility to understand how sexual minority individuals might strategically navigate decisions to reveal or conceal their sexual orientation to others. Given that this is the first direct application of this framework to the concealable stigma of sexual orientation, we used a qualitative research paradigm to solicit descriptions from sexual minority individuals of situations where they either revealed or concealed their sexual orientation to become more or less visible to others. This allowed us to test whether participants recognized the distinction between the two concepts and could provide descriptions of situations that fit this framework.

We found evidence that sexual minority individuals do indeed distinguish between concealment and invisibility, strategically revealing or concealing their sexual orientation to become visible or invisible in the pursuit of various goals. They may, for instance, reveal their sexual orientation to another sexual minority individual to become more visible as a potential friend or romantic partner, reveal their sexual orientation to a straight person to become invisible to unwanted romantic overtures, conceal their sexual orientation to be more visible during hiring or promotion decisions, or conceal their sexual orientation to become invisible to discrimination or unwanted questions.

Although we were able to collect responses that fit all four quadrants of this framework, the prompts elicited some responses that better fit another category. Specifically, participants were more likely to provide responses that fit their prompt in the reveal/visible condition and less likely to provide such responses in the reveal/invisible condition. Although the theoretical distinction between concealment and invisibility can help us to understand sexual minority people’s experiences, this distinction may not always be understood by or relevant to sexual minority individuals themselves. Alternatively, experiences of revealing to be visible may simply be more common and more salient than experiences of revealing to be invisible. Despite the lower rates of generating these latter responses, substantial numbers of participants were still able to do so, and described vivid experiences from their lives.

Certain trends emerge across the experiences and motives of participants. For one, affiliation was a primary motive in all response categories except revealing to be invisible. For many, interpersonal connection and group cohesion helped to guide decisions to reveal or conceal their sexual orientation: We saw that LGB people may reveal their sexual orientation to strengthen dyadic connections and affirm belonging with other LGBTQ+ people, and may conceal their sexual orientation to preserve relationships with, and avoid the prejudice of, those unlikely to accept them. These affiliation-motivated decisions primarily involved friends and coworkers. Indeed, strategic decisions about whether to disclose one’s sexual orientation may be particularly common in the workplace, where one may need to maintain social cohesion with others whom they might otherwise avoid, including those who are liable to respond negatively to disclosure of a minority sexual orientation. Correspondingly, we saw that job-related motives primarily emerged in participants who described concealing (rather than revealing) their sexual orientation.

Study 1 thus yields initial findings supporting a framework of disclosure decision-making that distinguishes between the acts of revealing and concealing and the goals of becoming socially visible or invisible. In Study 2, we attempt to confirm these findings with a separate sample of participants. Moreover, we conduct a preregistered analysis of participants’ prospective emotional experiences in the four quadrants of our theoretical framework, building upon exploratory Study 1 findings (reported in the Online Supplement) that disclosure and visibility elicit more positive retrospective emotion as well as specific emotions such as happiness and authenticity. Thus, Study 2 aims to better understand how anticipated emotions may influence the navigation of disclosure and invisibility.

## Study 2

In the previous study, we qualitatively demonstrated that LGB respondents’ decisions to reveal or conceal their sexual orientation show independence from whether they are seeking social visibility or invisibility in a given situation. Here, we quantitatively replicate this finding and furthermore assess the expected effects of the act of revealing or concealing one’s sexual orientation and the desire for social visibility or invisibility upon LGB people’s emotions.

### Method

The preregistration for this study can be found on OSF (https://osf.io/3fy7z/?view_only=64885060398048558ac7554d859fbdc7).

#### Participants

A priori power analysis using G*Power (Faul et al., 2009) showed that a sample size of *N* = 256 participants would offer adequate (80%) power to detect a small-to-medium effect (Cohen’s *f* = .18) in a two-by-two between-subjects ANOVA. We recruited a sample of 287 lesbian, gay and bisexual participants residing in the United States or Canada using Prolific. After excluding 34 participants who provided incomplete data and 12 participants who identified themselves as “Straight/Heterosexual” on our own demographics survey, we obtained a final sample of 241 participants (*M*_age_ = 33.77, *SD* = 10.49). A post hoc sensitivity analysis indicated that this sample size remains sufficient to detect a small-to-medium effect (Cohen’s *f* = .18) with adequate (80%) power. This final sample comprised 145 women (including one transgender woman), 74 men (including five transgender men and one nonbinary transgender man), 13 nonbinary participants, and nine participants of other genders. 147 participants reported their sexual orientation as bisexual, 61 as gay/lesbian/homosexual, 24 as pansexual, one as asexual, and seven as other sexual orientations; one participant did not indicate their sexual orientation.

#### Procedure

##### Imagined Situation Task

After participants provided informed consent and their Prolific ID, we instructed them to imagine a hypothetical social situation based on a randomly selected prompt. These prompts briefly described one-on-one social interactions in which the participant either reveals or conceals their sexual orientation to someone else with the goal of being either socially visible or invisible. This produced a two-by-two experimental design crossing behavior with goal, similar to the previous study. The prompts identified the other person in the social interaction as one of three randomly assigned roles: either a coworker, a stranger, or an online acquaintance. These roles were not of theoretical interest, but instead were chosen to ensure sampling across a greater breadth of possible relationships (Wells & Windschitl, 1999).^1^ Otherwise, the prompts included little detail, allowing participants to freely imagine the precise nature of the situation. The full prompts are available on OSF (https://osf.io/h5p4n/?view_only=e3e3ada2911d4ce39c5cee3bd1e11cac). For exploratory purposes and to ensure careful thought in response to the prompt, participants completed a free-response item in which they briefly described the situation they imagined before proceeding to the remainder of the experiment. We report an analysis of the correspondence between participants’ free responses and assigned condition in the Online Supplement.

##### Situation Ratings

Participants then reported the emotions they thought they would feel in the imagined situation. These emotion measures included an overall measure of positive versus negative emotion and specific measures for happiness, sadness, anxiety, authenticity, and acceptance. We selected these measures to capture the valence of expected emotions as well as to confirm the exploratory differences in emotional valence and in specific emotions such as happiness and authenticity suggested by the qualitative and quantitative results of Study 1 (quantitative analyses of emotions reported in Study 1 can be found in the Online Supplement). Participants responded to all emotion measures using 7-point Likert-type scales. For exploratory purposes (not pre-registered), participants also reported how much they thought they would like the other person in the imagined social situation, the ease of imagining a situation based on their prompt, and whether they thought a situation like the one they imagined could happen to them in real life.

### Results

For concision, we report only statistics for significant effects from our main analyses. We report complete statistics (including null effects, post hoc tests, and descriptive statistics) in the Online Supplement.

#### Emotion Measures

We conducted a two-way ANOVA analyzing the effects of behavior and goal on overall emotions. We conducted our analyses using the tidyverse (Wickham et al., 2019), sjstats (Lüdecke, 2022), rstatix (Kassambara, 2022), and effectsize (Ben-Shachar et al., 2024) packages for R (Version 4.1.1; R Core Team, 2021). We found a medium main effect of behavior, reveal versus conceal; *F*(1, 237) = 20.37, *p* < .001, η² = .08, and a large main effect of goal, visibility versus invisibility; *F*(1, 237) = 77.85, *p* < .001, η² = .25, such that participants thought they would feel more positive emotion when revealing their sexual orientation (compared with concealing) and when seeking visibility (compared with invisibility).

**Figure 6.**
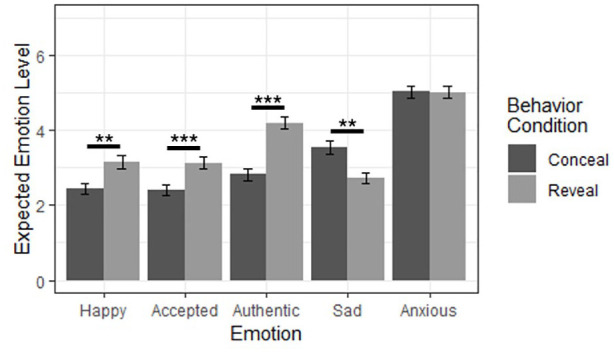
Main Effects of Behavior Condition on Specific Emotion Ratings. *Note.* **Bonferroni-corrected *p* < .01. *** Bonferroni-corrected *p* < .001. Error bars represent standard error.

**Figure 7:**
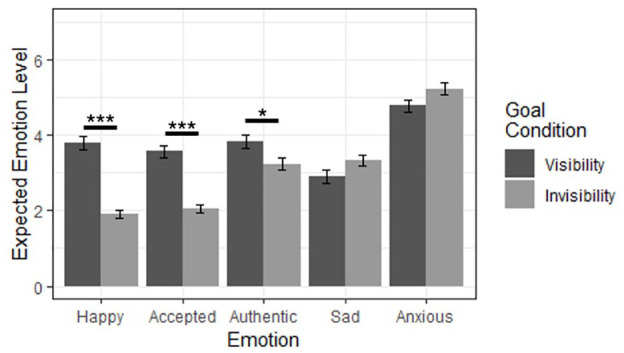
Main Effects of Goal Condition on Specific Emotion Ratings. *Note.* *Bonferroni-corrected *p* < .05. ***Bonferroni-corrected *p* < .001. Error bars represent standard error.

To test for effects on specific emotions, we conducted a two-way multivariate analysis of variance (MANOVA) of behavior and goal predicting the five specific emotion variables. We began with MANOVA as an initial omnibus test to avoid false positives due to multiple analyses, and likewise applied a Bonferroni correction to our post hoc analyses of variance (ANOVAs) to mitigate the type I error rate. We found strong main effects of behavior, Pillai’s *V* = .19, *F*(5, 233) = 10.97, *p* < .001, η² = .19, and goal, Pillai’s *V* = .33, *F*(5, 233) = 22.64, *p* < .001, η² = .33, as well as a moderate interaction effect, Pillai’s *V* = .06, *F*(5, 233) = 3.14, *p* = .009, η² = .06, indicating that the effects of behavior and goal differed across the specific emotions. To identify these differences, we performed five post hoc Bonferroni-corrected (α = .01) two-way ANOVAs. We found several main effects of behavior such that participants who imagined revealing (rather than concealing) their sexual orientation thought they would feel more happiness, acceptance, and authenticity, as well as less sadness (Figure 6). We likewise found several main effects of goal such that participants who imagined seeking visibility (rather than invisibility) thought that they would feel more happiness, acceptance, and authenticity (Figure 7). Following Bonferroni correction, we found no significant interaction effects.

#### Exploratory Measures

**Figure 8.**
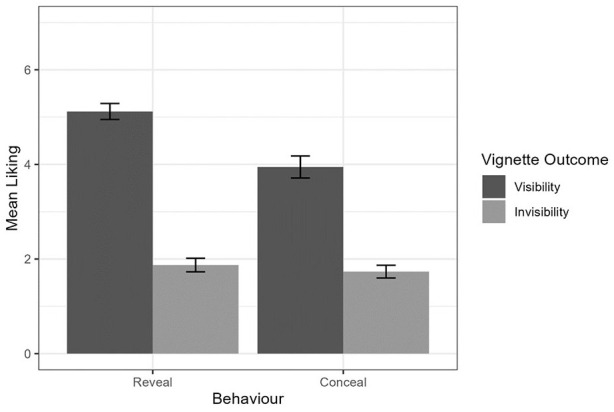
Effect of Behavior and Goal on Liking. *Note.* Error bars represent standard error.

We likewise ran three two-way ANOVAs examining the effects of behavior and goal on liking for the imagined interaction partner, ease of imagining the situation, and perceived likelihood that one might experience a similar situation in real life. For liking, we observed a medium main effect of behavior, *F*(1, 237) = 16.30, *p* < .001, η² = .06, and a large main effect of goal, *F*(1, 237) = 258.27, *p* < .001, η² = .52, such that participants believed they would like the other person more when revealing their sexual orientation and when seeking social visibility. We furthermore found a small interaction between behavior and goal, *F*(1, 237) = 9.23, *p* = .003, η² = .04, such that seeking invisibility attenuated the effect of behavior on liking for the other person (Figure 8).

Elsewhere, we found a small main effect of goal, *F*(1, 237) = 14.23, *p* < .001, η² = .06, on ease of imagining the situation, such that participants found it easier to imagine seeking invisibility than visibility, and a small interaction effect, *F*(1, 237) = 2.63, *p* = .017, η² = .02, such that participants found it easier to imagine concealing to be invisible compared with revealing to be invisible or concealing to be visible; no other ease ratings differed significantly. We also found small main effects of behavior, *F*(1, 237) = 6.95, *p* = .009, η² = .03, and goal, *F*(1, 237) = 10.74, *p* = .001, η² = .04, on perceived likelihood that the situation could occur in participants’ real lives, such that participants rated situations in which they imagined concealing their sexual orientation or seeking invisibility as more likely to occur. The mean perceived likelihood was above the midpoint of the scale (*M* = 5.27, *SD* = 1.90), as were all cell means for perceived likelihood (see Online Supplement).

### Discussion

Here, we examined whether participants would differentiate between concealment and invisibility when imagining a social situation and its emotional effects. We found multiple main effects of both behavior and goal upon expected emotion outcomes, which largely overlapped: Participants rated that they would feel happier, more authentic, and more accepted both when imagining revealing (rather than concealing) their sexual orientation and when seeking visibility (rather than invisibility). These separate effects of behavior and goal are additive, each contributing independently to participants’ rated emotions. That said, only behavior condition affected sadness ratings, with participants expecting greater sadness when concealing their sexual orientation, indicating that the emotional effects of concealing and seeking invisibility are not identical. Moreover, exploratory analyses revealed differences in the effects of behavior and goal upon liking for one’s imagined interaction partner. Seeking invisibility leads to similarly low liking regardless of whether one is revealing or concealing one’s sexual orientation, whereas when seeking visibility, participants rate their imagined interaction partner as more likable when revealing rather than concealing. Thus, invisibility and concealment show several distinct effects on downstream outcomes in an imagined interaction.

The effects of seeking invisibility upon emotion ratings reinforce Neel and Lassetter’s (2019) framing of interpersonal invisibility as stigmatizing: To be dismissed as irrelevant, even when one deliberately wishes to escape another person’s notice, is a negative emotional experience. This suggests that social invisibility may rarely be a preferred goal in social interactions, at least for this population, and that people instead seek invisibility primarily when it is anticipated to be the better option than visibility. This is consistent with Study 1 participants’ descriptions of seeking invisibility to escape being seen as affording an unwanted opportunity to others (e.g., disclosing their sexual orientation to avoid being seen as a prospective romantic partner by a straight person) or to avoid threat-based forms of stigma (e.g., concealing sexual orientation to avoid physical violence). This is further evidenced by the effects of desiring invisibility upon ratings of liking for one’s imagined interaction partner: Participants reported less liking for interaction partners to whom they imagined wanting to be invisible, suggesting that they imagined their interactions or the interaction partners themselves to be uncomfortable or off-putting in some way.

Furthermore, we found that situations in which participants concealed their sexual orientation were seen as more likely to occur in reality, and that situations in which participants sought invisibility were both easier to imagine and seen as more likely to occur. Situations that reflect a distinction between concealment and invisibility (i.e., revealing to be invisible and concealing to be visible) were less easy for participants to imagine than concealing to be invisible, but participants imagined these situations with similar ease compared with imagining revealing to be visible. Overall, mean ratings suggest that participants in all conditions tended to see the situations as plausible, further suggesting that the prompts—including those that describe revealing one’s sexual orientation to be invisible or concealing one’s sexual orientation to be visible—align with participants’ real-world experiences rather than representing wholly artificial situations.

We note that in contrast to Study 1, we analyzed participants’ ratings according to their assigned prompt rather than the content of their responses, to preserve true random assignment. Qualitative analysis of participants’ descriptions of the situations they imagined shows that the rate of definitively incongruent situations is consistently low across conditions (see Online Supplement, Table S3). However, the majority of participants did not include sufficient detail to judge whether or not the situation they imagined matched their assigned condition, perhaps due to the comparatively less thorough free-response probing used in this primarily quantitative study. We note that these participants did not report finding it more difficult to imagine a situation than their counterparts who provided more detailed responses (see Online Supplement), suggesting that responses with little detail are not simply an indicator that the participant struggled with their prompt. Nevertheless, in light of the response incongruence rates documented in Study 1, it may be that a number of these participants imagined situations that better corresponded to a different quadrant of our theoretical framework, due, for example, to a misconstrual of the prompt or to individual differences that make certain imagined situations more accessible than others. Thus, these analyses include an uncertain number of participants for whom our manipulation may not have functioned as expected.

## General Discussion

In this work, we used mixed methods to examine whether sexual minority individuals meaningfully distinguished between revealing or concealing one’s sexual orientation and social visibility or invisibility. Consistent with the assertion put forward by Neel and Lassetter (2019) that social invisibility is distinct from concealing a stigmatized identity, we found that participants described situations in which they revealed their sexual orientation to be invisible and situations in which they concealed their sexual orientation to be visible (Study 1), and that concealing one’s sexual orientation and seeking invisibility separately predict participants’ expected emotions in imagined social interactions. That said, we found that participants in Study 1 generated situation descriptions that matched their assigned condition more successfully (compared with the baseline rate of congruent responses across conditions) if they were prompted to remember revealing to become visible and less successfully if they were prompted to remember revealing to become invisible. Likewise, participants in Study 2 found it easier to imagine concealing to be invisible than to imagine concealing to be visible or revealing to be invisible, indicating that participants in both studies may associate concealing with invisibility more so than visibility. All the same, many Study 1 participants still described past experiences where they revealed to be invisible or concealed to be visible, and Study 2 participants rated the situations they imagined as plausible in all conditions, including those that prompted them to imagine concealing to be visible or revealing to be invisible. Thus, social invisibility does differ from identity concealment in ways that matter for LGB people’s real-world lived experiences.

Furthermore, these studies begin to elucidate the emotions associated with the different quadrants of our theoretical framework. In addition to the exploratory emotional outcomes from Study 1 discussed in the Online Supplement, Study 2 includes preregistered analyses of the overall positivity versus negativity of participants’ emotions in imagined situations and of various specific emotions. We find that concealment and invisibility are both imagined as negative emotional experiences that elicit a similar set of emotions, with the exception that seeking invisibility is not imagined to cause sadness, whereas participants imagined greater sadness when concealing rather than revealing their sexual orientation. That said, the magnitude of these emotion effects seems to differ, with seeking visibility (rather than invisibility) having a stronger effect on feelings of happiness and acceptance, and with revealing (rather than concealing) one’s sexual orientation having a stronger effect on feelings of acceptance and on overall positive or negative emotionality.

The present research also sheds light on the goals that motivate disclosure decisions. Participants who revealed their sexual orientations often did so for reasons relating to romantic motives, such as advertising their availability to potential partners or dissuading unwanted romantic interest. However, participants who concealed their sexual orientation often did so to maintain social cohesion in their workplace or with their kin, two relational contexts in which LGB people may not have the freedom to distance themselves from others who will mistreat them on the basis of their sexual orientation. Similarly, affiliation was a prevalent motive across several quadrants of our framework in Study 1, either to promote visibility as a means of continuing or strengthening a social relationship, or to achieve invisibility from negative attention in existing social relationships.

The prevalence of affiliation as a motive in Study 1 contextualizes differences in liking observed across quadrants of our theoretical framework in Study 2. Even when participants imagined concealing their sexual orientation to be visible to their interaction partner in Study 2, they still rated their interaction partner as more likable than in situations where participants imagined seeking invisibility. This reinforces that needing to conceal one’s sexual orientation from another person does not necessarily disqualify that person from being seen as likable (and, by extension, a desirable target for affiliation). Instead, LGB participants seem to accept that it is sometimes necessary to conceal one’s sexual orientation to maintain an otherwise desirable social interaction or relationship. We note that, whereas affiliation also often motivated concealing one’s sexual orientation to be invisible in Study 1, this tended to occur in preexisting (rather than new) friendships or to maintain social cohesion in the workplace (rather than cultivate friendships with liked others). Given that the imagined interaction partners in Study 2 were either strangers, coworkers, or acquaintances (rather than existing friends), Study 2 participants may not have seen a desire for invisibility and a desire for affiliation as compatible in this specific relationship context, and thus rated their imagined interaction partners as less likable .

Note that we also see evidence of heterogeneity in social invisibility experiences across different minority sexual orientations. Unlike gay men and lesbians, bisexual people may form romantic relationships with other-gender partners and can thus become romantically or sexually involved with straight people. In Study 1, some bisexual participants described revealing their sexual orientation to assess whether an other-gender partner is homophobic, or concealing their sexual orientation out of concern for how an other-gender partner might react. Bisexual respondents futhermore expressed concerns about the erasure of their bisexual identity or the existence of biphobia among gay men and lesbians. This is not to suggest that bisexual people share no common experiences with gay men and lesbians—indeed, most motives and experiences emerged across minority sexual orientations. Nevertheless, the experiences of sexual minority people with different identities are not interchangeable, and consideration must be given to how a person’s specific identity may shape the situations they encounter and the decisions they make.

In a similar vein, this work is limited by its specific focus on sexual orientation without direct consideration of its intersection with other identities, including transgender and gender nonconforming (TGNC) identities. Our specific recruitment of sexual minority participants did yield a high proportion of TGNC participants in our Study 1 sample relative to the general population: 12.5% of our participants reported their gender with descriptors other than or in addition to “man” or “woman,” whereas recent estimates of the prevalence of TGNC individuals in the general population range from 0.1% to 2.7% (Goodman et al., 2019; Nolan et al., 2019). Nevertheless, although some participants gave responses that illustrate the complexities of simultaneously concealing one’s sexual orientation and gender, our data does not account in-depth for these situations. It also does not provide insight into the various forms that concealment of TGNC identity can take when not considering sexual orientation. Likewise, the present work does not directly examine the experiences of sexual minority individuals in relation to racial or ethnic identity. However, in light of phenomena such as sexual racism within the LGBTQ+ community (e.g., Callander et al., 2015; Han & Choi, 2018) and the interaction between stereotypes pertaining to race and sexual orientation (Remedios et al., 2011), the specific experiences of sexual minority people of color during reveal/conceal decisions merit further, more targeted research, as do LGB people of color’s experiences of invisibility in queer social contexts. Future research may therefore examine these phenomena with specific attention to intersections between sexual minority status and other stigmatized identities.

Future work may likewise seek to extend these findings to other concealable stigmatized identities (e.g., members of stigmatized religious groups, people with mental illnesses). Some of the experiences identified in this work may be broadly applicable across concealable stigmas, such as electing to hide a stigmatized identity at work to remain visible to professional opportunities or revealing a shared stigma to pursue friendship with an ingroup member. Moreover, other experiences that emerged rarely or not at all in the present research may be much more common for people with other concealable stigmas, such as hiding one’s identity from a romantic partner to protect one’s relationship. Other groups may likewise face distinctive situations specific to their own needs and experiences, such as people with concealable disabilities disclosing their identity so as not to be called upon to engage in an activity that is taxing or impossible for them to perform. Thus, further studies should examine how social invisibility shapes disclosure decisions for members of other concealable stigmatized groups, both to further test this theoretical framework and to better understand the experiences of these social groups.

Finally, future research can expand upon this theory by identifying the antecedents of the desire for invisibility (vs. visibility) that shape strategic disclosure decisions. For instance, it may be that people desire invisibility when interacting with another person whose motives or goals are incompatible with their own, to avoid being viewed as affording an opportunity for the pursuit of these motives and goals (e.g., disclosing a minority sexual identity to avoid a straight person’s sexual interest). It may also be that targets desire invisibility when they anticipate that visibility would entail being seen as a threat, leading them to seek invisibility to be passively ignored rather than subjected to active negative attention. Similarly, future research may consider the antecedents and consequences of active concealment (vs. passive nondisclosure) in relation to visibility and invisibility, as well as examine how these goals may modulate the effects of these behaviors on well-being and emotions. For instance, the emotional effects of invisibility may differ in quality and magnitude when that invisibility is deliberately sought compared with when a person who wishes to be visible is nevertheless dismissed as irrelevant. Empirically examining these and other potential factors that may elicit a desire for invisibility would help to further elucidate the strategic use of disclosure and concealment as a means of managing one’s social visibility.

## Conclusion

The present research explores identity disclosure decisions in sexual minority individuals as a possible means of deliberately managing how others value and pay attention to them, in keeping with the affordance-management theory of interpersonal invisibility. We found that participants reflecting on their own experiences could describe situations where they either revealed or concealed their sexual orientation to become either visible or invisible to others, and did so in service of various goals. Participants rating their expected emotional outcomes in hypothetical situations likewise showed separate effects of concealment (vs. disclosure) and seeking invisibility (vs. visibility) upon their expected emotions. We thus find direct evidence for the applicability of the affordance-management theory of interpersonal invisibility to concealable stigmatized identities and the effects of concealment and visibility. Furthermore, we expand upon the existing literature on concealment of sexual orientation by identifying a framework that can account for disclosure decisions in specific situations and social interactions. We also shed light on the motives associated with disclosure decisions and with aims to become socially visible or invisible, as well as the emotions participants expect to follow from such decisions. Additional research may examine specific experiences at the intersection of sexual orientation and other identities, such as racial or ethnic identity or TGNC identity, allowing us to further advance understanding of sexual minority individuals’ ongoing decisions to reveal or conceal their sexual orientation.

## Supplemental Material

sj-docx-1-psp-10.1177_01461672241313269 – Supplemental material for Sexual Orientation Disclosure and Strategic Navigation of Interpersonal Invisibility Supplemental material, sj-docx-1-psp-10.1177_01461672241313269 for Sexual Orientation Disclosure and Strategic Navigation of Interpersonal Invisibility by Emily Schwartzman and Rebecca Neel in Personality and Social Psychology Bulletin

## Notes

1. ANOVAs examining the effects of the imagined interaction partner revealed a small effect on sadness ratings, *F*(1, 237) = 4.82, *p* = .009, η² = .04, such that participants reported greater sadness in interactions with online acquaintances compared with strangers. Otherwise, the interaction partner did not significantly affect ratings.
