# Supplementary material for: Sexual Orientation Disclosure and Strategic Navigation of Interpersonal Invisibility
Source: Pers Soc Psychol Bull. 2025 Jan 24;52(5):1341–55. doi: 10.1177/01461672241313269 (PMC13022016; doi:10.1177/01461672241313269)
Supplement: sj-docx-1-psp-10.1177_01461672241313269 – Supplemental material for Sexual Orientation Disclosure and Strategic Navigation of Interpersonal Invisibility [file sj-docx-1-psp-10.1177_01461672241313269.docx]

**Study 1**

**Analytic Approach**

Participants’ answers in the free-response task were analyzed with qualitative coding by two trained research assistants. We developed codes through a combination of deductive and inductive coding: We began with a set of deductive a priori codes, and the coding team met weekly to discuss edge cases and iteratively modify the codebook to refine existing codes and add new codes as needed. Responses tended to be brief (combining a given participant’s answers for the four questions typically yielded a paragraph or less of text), so we value-coded each participant’s responses in their entirety rather than applying tags to distinct units of text. A brief overview of the codes follows. A full codebook containing the final list of codes, values, and criteria can be found on page 2 of this supplement. Upon completion of coding, all codes showed acceptable interrater reliability (i.e., Cohen’s Kappa ≥ 0.8).

Raters categorized responses by the behavior (revealing or concealing sexual orientation) and objective (visibility or invisibility) participants described. During coding, raters were blind to participants’ actual assigned condition and prompt. Raters also coded for the apparent motive(s), if any, of the participant and of the person with whom the participant described interacting. We adapted an initial a priori set of values for the motive codes from the Fundamental Social Motives Inventory (Neel et al., 2016). The Fundamental Motives Framework is a widely-used approach, and describes a set of social motivations that humans have developed to manage the recurrent challenges and opportunities of social living. The motives include protecting oneself from harm, avoiding disease, affiliating with others, attaining status, finding and keeping mates, and caring for kin (Kenrick et al., 2010; Schaller et al., 2017). Other work has similarly asked people to report on their experiences and then coded responses for congruence with fundamental motives (e.g., Krems et al., 2017). For the current project, we expanded this set of motives, based on our data, to include employment (the motive to seek and retain a job and carry out duties related to one’s work), avoiding unwanted romantic interest (a counterpart to the motive to seek mates, representing the motive to deter undesired romantic and sexual attention; see Pirlott & Neuberg, 2014), authenticity (the motive to be oneself and be open about one’s identity), and hiding (concealing one’s sexual orientation because one feels unready to come out). Motive codes were not mutually exclusive and more than one motive could be assigned to the same response. Raters also coded for the context in which the situations took place, and whether the participant’s interaction with the other person was digitally mediated.

Raters also applied several data quality codes, including whether participants were able to respond to the prompt, whether they described a single specific incident or a generalized repeating occurrence, and whether participants explicitly described themselves as straight or heterosexual in their responses (to identify participants who provided inaccurate demographic information in Prolific’s prescreening questionnaire and exclude them from analysis).

**Qualitative Codes, Values, and Criteria**

Our qualitative codebook is reproduced below. The codebook is organized by codes (i.e., coded variables), featuring a description of each code and its possible values.

| **Code:** | **Reveal/Conceal_resp** |
| --- | --- |
|  | Without consulting the participant's assigned condition, do they describe revealing their sexual orientation (e.g., disclosing or otherwise making the info available to someone else), or concealing their sexual orientation (misleading someone else, withholding relevant information, etc.). Where someone describes a situation where their understanding of their sexual orientation changes over time (e.g., from when they previously identified as straight), apply the reveal or conceal code that is congruent with their understanding of their sexual orientation when the situation took place.  This can also include concealing one's own gender identity to avoid having a relationship with another person be read as queer (applicable to trans & genderqueer respondents) |
| **Value** | **Description** |
| reveal | The participant explicitly disclosed their sexual orientation, shared info that would allow the other person to infer their orientation or their membership to the queer community, etc. Apart from verbal behaviour, this can also include deliberate appearance cues (e.g., deliberately wearing or displaying rainbow/Pride items, self-presenting in a manner meant to convey LGB status). |
| conceal | The participant withheld information about their orientation, provided information  that was misleading/inaccurate or vague, etc. This can also include deliberately modifying one's personal appearance to be less visibly LGB. |
| conceal&reveal | This covers, among other things, situations where someone outs themself as LGB but otherwise hides or misleads about their precise orientation within that umbrella. |
| na | The conceal/reveal distinction is not applicable to the situation the participant describes. |

| **Code:** | **Visible/Invisible_resp** |
| --- | --- |
|  | Without consulting the participant's assigned condition, do they attempt to make themselves  visible/relevant to the other person's ostensible goals, or do they attempt to make themselves invisible/irrelevant? Do they aim to have the other person pay attention to them, or ignore them? Where there is a conflict between visibility for an immediate goal and invisibility for a later, latent goal, the response should be coded as visible (unless the respondent specifically describes thinking of the later latent goal in their reasoning for their behaviour).  The values within this code are mutually exclusive: Only one value can apply to each response. |
| **Value** | **Description** |
| visible | The participant's behaviour was intended to increase/maintain their visibility, that they would be seen as relevant to the other person's goals or be attended to in some way. |
| invisible | The participant's behaviour was intended to make them invisible/irrelevant to the other person's goals, or to otherwise evade another person's notice. |
| na | The visible/invisible distinction is not applicable to the situation the participant describes. |
|  |  |

| **Code:** | **Participant motive** |
| --- | --- |
|  | What fundamental social motive (if any) was the participant seeking to fulfill through their behaviour? These motives are drawn from Neel et al. (2016) and informed by Kenrick et al. (2010), but some may not appear within the data. These values **can co-occur**. |
| **Value** | **Description** |
| aff | Affiliation. Motivates forming and maintaining social bonds with others, outside of kin/family or mates/romantic partners. Making and retaining friendships, forming groups and ensuring those groups get along, and avoiding being socially excluded all reflect an affiliation motive. Rule in this motive when participant explicitly describes wanting to keep a conversation going/wanting to get along as part of their motive for responding a certain way.  To be classed as affiliation, a response must exhibit or imply one of the above criteria; if a conversation is happening, the motive is NA unless this or another motive is apparent.  This motive includes disclosing to show belonging in an LGBTQ+ space (affiliation with a relevant group). |
| dis | Disease Avoidance. Motivates avoiding disease/contagion from other people. |
| kin | Kin Care. Motivates caring for and maintaining good relationships with family members.  Rule in if a participant expresses explicit concern over a family member being upset, disturbed, angry, etc. |
| mate_avoid | Avoid Unwanted Romantic Attention (added; referred to as Mate Avoidance during coding). Participant is striving to be ignored by another person seeking a romantic/sexual partner. |
| mate_ret | Mate Retention. Motivates efforts to maintain an existing romantic/sexual partnership, as well as generates concerns about infidelity or potentially losing one's partner. Working to ensure a partner's ongoing investment in the relationship and avoid breakups/infidelity reflect a mate retention motive. |
| mate_seek | Mate Seeking. Motivates searching for potential romantic/sexual partners and attempting to  initiate romantic/sexual relationships. |
| self_prot | Self Protection. Motivates the avoidance of danger posed by other people, outside of dangers  pertaining to infection/contagion. Prototypical example is physical attack by others. |
| stat | Status. Motivates seeking elevated positions within hierarchies, avoiding losing the status one  holds, and seeking to lead/be admired by others. Distinguished from affiliation by the desire to be superior to other people in some way. |
| job | Job/employment. Data-driven motive, not part of FSM literature. Motivates seeking employment and avoiding losing one's existing job. Also covers carrying out responsibilities immediately related to one's job. |
| auth | Authenticity. Behaving in a way that allows one to be open and honest about their identity, and/or accepted by others for who they are.  Edge case: revealing sexual orientation to make a point to someone who is being bigoted or otherwise being exposed to homophobic views.  Not included under authenticity: disclosing to show belonging in a queer space. This is externally motivated and falls under affiliation instead. |
| Hide | Hiding/remaining in the closet. Concealing for no particular reason other than that the participant is not ready to come out to the other person. |
| Other | Another motive/goal. Highlight this cell and add a tentative code in the Notes column. |
| Na | No motive/goal is discernible. |

| **Code:** | **Other-person motive** |
| --- | --- |
|  | What fundamental social motive (if any) seemed to be active/relevant for the other person/people involved in the situation the participant describes.  These motives are drawn from Neel et al. (2016) and informed by Kenrick et al. (2010), but some may not appear within the data. These values **can co-occur**. |
| **Value** | **Description** |
| aff | Affiliation. Motivates forming and maintaining social bonds with others, outside of kin/family or mates/romantic partners. Making and retaining friendships, looking after the well-being of friends, forming groups and ensuring those groups get along, and avoiding being socially excluded all reflect an affiliation motive. To be classed as affiliation, a response must exhibit or imply one of the above criteria. If a conversation is happening, the motive is NA unless this or another motive is apparent. |
| dis | Disease Avoidance. Motivates avoiding disease/contagion from other people. |
| kin | Kin Care. Motivates caring for and maintaining good relationships with family members. |
| mate_ret | Mate Retention. Motivates efforts to maintain an existing romantic/sexual partnership, as well as generates concerns about infidelity or potentially losing one's partner. Working to ensure a partner's ongoing investment in the relationship and avoid breakups/infidelity reflect a mate retention motive. |
| mate_seek | Mate Seeking. Motivates searching for potential romantic/sexual partners and attempting to  initiate romantic/sexual relationships. |
| self_prot | Self Protection. Motivates the avoidance of danger posed by other people, outside of dangers  pertaining to infection/contagion. Prototypical example is physical attack by others. |
| stat | Status. Motivates seeking elevated positions within hierarchies, avoiding losing the status one  holds, and seeking to lead/be admired by others. Distinguished from affiliation by the desire to be superior to other people in some way. |
| job | Job/employment. Data-driven motive, not part of FSM literature. Motivates seeking employment and avoiding losing one's existing job. Also covers carrying out responsibilities immediately related to one's job. |
| other | Another motive/goal. Highlight this cell and add a tentative code in the Notes column. |
| na | No motive/goal is discernible. |

| **Code:** | **Context** |
| --- | --- |
|  | In what location/context did the situation take place? |
| **Value** | **Description** |
| home.a | Situation takes place in the participant's household/living space. If both home.a and home.b are true (i.e., if participant and other person live together), then apply home.a. |
| home.b | Situation takes place in the other person's household/living space. |
| home.c | Situation takes place in a household/living space not belonging to either party. |
| home.x | Situation takes place in a household/living space, but not clear whose. |
| work | Situation takes place at work (including prospective workplaces, e.g., for job interviews). |
| school | Situation takes place at a school/university/place of learning. Note that this only refers to schools/universities where the respondent **is a student**. For instance, if the respondent is a teacher and describes something that took place in the classroom, then the appropriate value is "work". |
| public | Situation takes place in a public space (outdoors, shop, restaurant, etc.) where the respondent is not an employee or student. |
| social_media | The interaction was digital and took place on social media. |
| na | The context isn't clear from the participant's response. If a specific context is implied (but not certain from the response), add to the Notes column "Implied context: [relevant code]", e.g., Implied context: school. |
| other | Another context. Highlight this cell and add a tentative code in the Notes column. |

| **Code:** | **Digital** |
| --- | --- |
|  | Does the interaction take place digitally while also primarily reflecting another context (e.g., Zoom call for school or work)? |
| **Value** | **Description** |
| 1 | Situation is digital. |
| 0 | Situation is not digital or there is no clear indicator either way. |

| **Code:** | **Specific incident?** |
| --- | --- |
|  | Does the participant describe a specific incident, or a general/recurring event? Data quality check; numeric codes. |
| **Value** | **Description** |
| 2 | Specific incident: the described situation happened only once, or the participant describes a single, particular instance of an event that happened multiple times. |
| 1 | Non-specific incident. The participant generalizes about something that happened multiple times. |
| 0 | The participant does not describe any incident/event. |

| **Code:** | **Unable to respond?** |
| --- | --- |
|  | Does the participant describe being unable to generate a response/example, or otherwise not understanding the prompt or instructions? |
| **Value** | **Description** |
| 2 | The participant explicitly states that they do not have an example. |
| 1 | The participant states that they do not understand the prompt/instructions. |
| 0 | The participant provides a description in response to the prompt |

| **Code:** | **Straight?** |
| --- | --- |
|  | **Within the response**, does the participant indicate that they actually identify as straight/ heterosexual (i.e., does their response contradict the screening information)?  This refers to a participants' actual underlying identity, not to an identity they may outwardly convey in the interest of concealment.  This has some degree of redundancy with the "Demo S.O." code below, but is included separately because participants may misrepresent themselves in the demographics section. This allows us to cast a slightly wider net for identifying straight respondents. |
| **Value** | **Description** |
| 1 | Straight: the participant describes themselves as straight/heterosexual or otherwise indicates that they are attracted to other-gender people **and** not attracted to people who share their own gender.  **Allusions to other-gender attraction/partners are not enough on their own** as these are also applicable to bisexual respondents. |
| 0 | Not straight: the participant's response does not indicate that they are straight/heterosexual. |

| **Code:** | **Demo S.O.** |
| --- | --- |
|  | Demographic sexual orientation--copy in the sexual orientation the participant reports in the demographic questionnaire. |

| **Code:** | **Reveal/Conceal actual** |
| --- | --- |
|  | After completing all preceding coding, copy in the participant's assigned behavior condition. |

| **Code:** | **RC_Match?** |
| --- | --- |
|  | Does the value for Reveal/Conceal_actual match the value for Reveal/Conceal_resp (the value based on their response alone)? Binary. |
| **Value** | **Description** |
| 1 | Match; both values are "reveal" or both values are "conceal" |
| 0 | Does not match. |

| **Code:** | **Visible/Invisible_actual** |
| --- | --- |
|  | After completing all other preceding coding, copy in the participant's assigned outcome condition. |

| **Code:** | **VI_Match?** |
| --- | --- |
|  | Does the value for Visible/Invisible_actual match the value for Visible/Invisible_resp (the value based on their response alone)? Binary. |
| **Value** | **Description** |
| 1 | Match; both values are "visible" or both values are "invisible" |
| 0 | Does not match. |
|  |  |

**Additional Statistics and Analyses**

***Ease of Responding***

**Table S1A**

*ANOVA Outputs for Effects of Assigned Condition on Ease of Responding*

|  |  |  |  |  |  |  |  |  |  |  |  |  |  |
| --- | --- | --- | --- | --- | --- | --- | --- | --- | --- | --- | --- | --- | --- |
|  | | **Sum of Squares** | | **df** | | **Mean Square** | | **F** | | **p** | | **η²** | |
| Behavior |  | 6.75 |  | 1 |  | 6.75 |  | 2.18 |  | .142 |  | .01 |  |
| Objective |  | 0.10 |  | 1 |  | 0.10 |  | 0.03 |  | .858 |  | .00 |  |
| Behavior ✻ Objective |  | 3.10 |  | 1 |  | 3.10 |  | 1.00 |  | .319 |  | .01 |  |
| Residuals |  | 515.46 |  | 166 |  | 3.11 |  |  |  |  |  |  |  |
|  | | | | | | | | | | | | | |

**Table S1B**

*Cell Means, Standard Deviations, and n Values for Ease of Responding*

| Behavior | Outcome | *M* | *SD* | *n* |
| --- | --- | --- | --- | --- |
| Reveal | Visible | 4.89 | 1.92 | 46 |
| Reveal | Invisible | 4.58 | 2.01 | 40 |
| Conceal | Visible | 5.03 | 1.61 | 40 |
| Conceal | Invisible | 5.25 | 1.45 | 44 |

***Reported Emotions by Response Type***

We conducted a two-way between-subjects ANOVA to examine how the behavior (reveal vs. conceal) and desired outcome (visibility vs. invisibility) participants described influenced their overall positive or negative emotional experience in that situation. Cell sizes were uneven because we grouped responses according to coded reveal/conceal and visibility/invisibility, rather than the conditions to which participants were assigned. Levene’s test revealed that the data was heteroscedastic, *F*(3,166) = 5.85, *p* < .001, so we applied White’s heteroscedasticity correction when computing standard errors. Participants experienced more positive emotion when revealing their sexual orientation (*M* = 4.59, *SD* = 1.21) than when concealing it (*M* = 3.09, *SD* = 1.89; *F*(1,166) = 25.93, *p* < .001), and when seeking visibility (*M* = 4.32, *SD* = 1.80) than when seeking invisibility (*M* = 3.17, *SD* = 1.43; *F*(1,166) = 7.65, *p* < .001). Behavior and outcome did not interact, *F*(1,166) = 1.93, *p* = .16. Nevertheless, the significant main effects provide initial evidence that concealment and invisibility have distinct emotional consequences.

Our analyses of reported specific emotions (Tables S2A-S2E) likewise identified separate effects of behavior (reveal vs. conceal) and goal (visibility vs. invisibility) on emotions, again based on participants’ responses rather than random assignment. Participants who described revealing their sexual orientation reported less shame and more relief, authenticity, and happiness. Participants who described seeking visibility reported more authenticity and happiness. Behavior and goal did not interact in any analyses, and the conditions showed no significant differences in anxiety.

**Table S2A**

*Complete Statistics for Shame ANOVA*

|  | | ***df*** | | ***F*** | | | ***p*** | | | **η²** | | | |
| --- | --- | --- | --- | --- | --- | --- | --- | --- | --- | --- | --- | --- | --- |
| Behavior |  | 1 |  |  | 28.08 |  | | < .001 |  | | .15 |  |  |
| Goal |  | 1 |  |  | 1.33 |  | | .250 |  | | .01 |  |  |
| Behavior ✻ Goal |  | 1 |  |  | 0.04 |  | | .850 |  | | .00 |  |  |
| Residual |  | 166 |  |  |  |  | |  |  | |  |  |  |

*Note.* Levene’s test indicated unequal variances (*F*(3, 166) = 4.61, *p* = .004), so we applied White’s heteroskedacity correction to this analysis.

**Table S2B**

*Complete Statistics for Relief ANOVA*

|  | | ***df*** | | ***F*** | | | ***p*** | | | **η²** | | | |
| --- | --- | --- | --- | --- | --- | --- | --- | --- | --- | --- | --- | --- | --- |
| Behavior |  | 1 |  |  | 23.08 |  | | < .001 |  | | .12 |  |  |
| Goal |  | 1 |  |  | 0.02 |  | | .904 |  | | .00 |  |  |
| Behavior ✻ Goal |  | 1 |  |  | 0.26 |  | | .614 |  | | .00 |  |  |
| Residual |  | 166 |  |  |  |  | |  |  | |  |  |  |

**Table S2C**

*Complete Statistics for Authenticity ANOVA*

|  | | ***df*** | | ***F*** | | | ***p*** | | | **η²** | | | |
| --- | --- | --- | --- | --- | --- | --- | --- | --- | --- | --- | --- | --- | --- |
| Behavior |  | 1 |  |  | 96.64 |  | | < .001 |  | | .42 |  |  |
| Goal |  | 1 |  |  | 10.45 |  | | .001 |  | | .08 |  |  |
| Behavior ✻ Goal |  | 1 |  |  | 0.04 |  | | .834 |  | | .00 |  |  |
| Residual |  | 166 |  |  |  |  | |  |  | |  |  |  |

*Note.* Levene’s test indicated unequal variances (*F*(3, 166) = 5.49, *p* = .001), so we applied White’s heteroskedacity correction to this analysis.

**Table S2D**

*Complete Statistics for Happiness ANOVA*

|  | | ***df*** | | ***F*** | | | ***p*** | | | **η²** | | | |
| --- | --- | --- | --- | --- | --- | --- | --- | --- | --- | --- | --- | --- | --- |
| Behavior |  | 1 |  |  | 19.78 |  | | < .001 |  | | .11 |  |  |
| Goal |  | 1 |  |  | 16.85 |  | | < .001 |  | | .09 |  |  |
| Behavior ✻ Goal |  | 1 |  |  | 0.00 |  | | .986 |  | | .00 |  |  |
| Residual |  | 166 |  |  |  |  | |  |  | |  |  |  |

**Table S2E**

*Complete Statistics for Anxiety ANOVA*

|  | | ***df*** | | ***F*** | | | ***p*** | | | **η²** | | | |
| --- | --- | --- | --- | --- | --- | --- | --- | --- | --- | --- | --- | --- | --- |
| Behavior |  | 1 |  |  | 3.11 |  | | .080 |  | | .02 |  |  |
| Goal |  | 1 |  |  | 0.23 |  | | .630 |  | | .00 |  |  |
| Behavior ✻ Goal |  | 1 |  |  | 0.03 |  | | .857 |  | | .00 |  |  |
| Residual |  | 166 |  |  |  |  | |  |  | |  |  |  |

**Study 2**

**Free Responses By Assigned Condition**

To verify whether participants in Study 2 imagined situations that aligned with their assigned condition, two independent raters (a trained research assistant and one of the authors) read and qualitatively coded each participant’s answer to the free-response item, “Please briefly describe the situation you imagined,” while blind to participants’ assigned condition. Raters coded the behavior and goal described in each response using the criteria developed for Study 1, reported in the above codebook. Interrater reliability was adequate, κ = .75 for behavior and κ = .72 for goal.

Of the 241 responses, a large proportion (*n* = 128) could not be assigned a behavior code and/or a goal code due to a lack of sufficient detail in the participant’s description. This may be due to the phrasing of the free-response item and its solicitation of a brief description, which was included to reduce the burden placed on participants in an otherwise quantitative study.

Based on these codes, we evaluated congruence between the prompt received and the response provided, with responses classified as insufficiently detailed as needed (Table S3). We conducted a chi-square test in R (Version 4.1.1; R Core Team, 2021) using the rstatix package (Kassambara, 2022) and the lsr package (Navarro, 2015) and found that the rate of congruence varied by assigned prompt (χ^2^(6) = 21.16, *p* = .001, Cramer’s *V* = .21). Post hoc analysis of residuals with Bonferroni-corrected *p* values showed that, compared to the overall rate of congruent, incongruent, and insufficiently detailed answers pooled across prompt conditions, participants assigned to the reveal/visible prompt were more likely to provide responses congruent with the prompt (*p* = .022) and less likely to provide insufficiently detailed responses (*p* = .023). In contrast, participants assigned to the conceal/invisible prompt were comparatively less likely to provide congruent responses (*p* = .017) and more likely to provide insufficiently detailed responses (*p* = .005). Congruence rates for the reveal/invisible and conceal/visible prompts did not differ from the overall frequency of congruent responses (all *p*s > .999), and all four prompts elicited incongruent responses at similar rates (all *p*s > .999).

Overall, the prevalence of insufficiently detailed free responses makes it difficult to conclude whether participants’ imagined situations generally corresponded to their assigned prompt. We cannot distinguish participants who provided insufficiently detailed responses because they poorly or improperly understood their assigned prompt from participants who imagined an appropriate situation but provided insufficiently detailed responses because we did not probe for in-depth descriptions. That said, we do note that, compared to those who provided detailed responses, participants who provided insufficiently detailed responses found it similarly easy to imagine a situation in response to the prompt, t(238.83) = 1.66, p = .098, Cohen’s d = 0.21, and rated their imagined situations as similarly plausible in real life, t(237.29) = 1.17, p = .245, Cohen’s d = 0.15. At the very least, we cannot conclude that the participants who provided insufficiently detailed responses struggled more with the task or found their imagined situations less believable.

**Table S3**

*Contingency Table for Response Type by Condition in Study 2*

| Assigned Condition | Congruent Responses | Incongruent Responses | Insufficiently Detailed Responses |
| --- | --- | --- | --- |
| Reveal, Visible | 35 | 3 | 21 |
| Reveal, Invisible | 32 | 2 | 29 |
| Conceal, Visible | 18 | 5 | 32 |
| Conceal, Invisible | 16 | 2 | 46 |

**Additional Statistics**

***Emotion Measures***

**Table S4A**

*Complete Statistics for Overall Emotion ANOVA*

|  | | **Sum of Squares** | | ***df*** | | **Mean Square** | | ***F*** | | ***p*** | | **η²** | |
| --- | --- | --- | --- | --- | --- | --- | --- | --- | --- | --- | --- | --- | --- |
| Behavior |  | 35.18 |  | 1 |  | 35.18 |  | 20.37 |  | < .001 |  | .08 |  |
| Goal |  | 134.45 |  | 1 |  | 134.45 |  | 77.85 |  | < .001 |  | .25 |  |
| Behavior ✻ Goal |  | 2.92 |  | 1 |  | 2.92 |  | 1.69 |  | .195 |  | .00 |  |
| Residual |  | 409.30 |  | 237 |  | 1.73 |  |  |  |  |  |  |  |
|  | | | | | | | | | | | | | |

**Table S4B**

*Cell Means, Standard Deviations, and n Values for Overall Emotion ANOVA*

| Behavior | Outcome | *M* | *SD* | *n* |
| --- | --- | --- | --- | --- |
| Reveal | Visible | 4.24 | 1.67 | 59 |
| Reveal | Invisible | 2.52 | 1.24 | 63 |
| Conceal | Visible | 3.27 | 1.42 | 55 |
| Conceal | Invisible | 2.00 | 0.82 | 64 |

**Table S5**

*Complete Statistics for Specific Emotion MANOVA*

|  | | **Pillai’s *V*** | | **Numerator *df*** | | **Denominator *df*** | ***F*** | | ***p*** | | **η²** | |
| --- | --- | --- | --- | --- | --- | --- | --- | --- | --- | --- | --- | --- |
| Behavior |  | .19 |  | 5 |  | 233 | 10.97 |  | < .001 |  | .19 |  |
| Goal |  | .33 |  | 5 |  | 233 | 22.64 |  | < .001 |  | .33 |  |
| Behavior ✻ Goal |  | .06 |  | 5 |  | 233 | 3.14 |  | .009 |  | .06 |  |

**Table S6A**

*Complete Statistics for Post Hoc Happiness ANOVA*

|  | | **Sum of Squares** | | ***df*** | | **Mean Square** | | ***F*** | | ***p*** | | **η²** | |
| --- | --- | --- | --- | --- | --- | --- | --- | --- | --- | --- | --- | --- | --- |
| Behavior |  | 29.01 |  | 1 |  | 29.01 |  | 20.37 |  | < .001 |  | .06 |  |
| Goal |  | 209.96 |  | 1 |  | 209.96 |  | 77.85 |  | < .001 |  | .30 |  |
| Behavior ✻ Goal |  | 5.17 |  | 1 |  | 2.48 |  | 1.69 |  | .117 |  | .01 |  |
| Residual |  | 494.90 |  | 237 |  | 2.09 |  |  |  |  |  |  |  |
|  | | | | | | | | | | | | | |

**Table S6B**

*Complete Statistics for Post Hoc Sadness ANOVA*

|  | | **Sum of Squares** | | ***df*** | | **Mean Square** | | ***F*** | | ***p*** | | **η²** | |
| --- | --- | --- | --- | --- | --- | --- | --- | --- | --- | --- | --- | --- | --- |
| Behavior |  | 40.16 |  | 1 |  | 40.16 |  | 13.41 |  | < .001 |  | .05 |  |
| Goal |  | 9.70 |  | 1 |  | 9.70 |  | 3.24 |  | .073 |  | .01 |  |
| Behavior ✻ Goal |  | 0.73 |  | 1 |  | 0.73 |  | 0.24 |  | .622 |  | .00 |  |
| Residual |  | 709.67 |  | 237 |  | 2.99 |  |  |  |  |  |  |  |
|  | | | | | | | | | | | | | |

**Table S6C**

*Complete Statistics for Post Hoc Authenticity ANOVA*

|  | | **Sum of Squares** | | ***df*** | | **Mean Square** | | ***F*** | | ***p*** | | **η²** | |
| --- | --- | --- | --- | --- | --- | --- | --- | --- | --- | --- | --- | --- | --- |
| Behavior |  | 114.99 |  | 1 |  | 114.99 |  | 40.60 |  | < .001 |  | .15 |  |
| Goal |  | 19.89 |  | 1 |  | 19.89 |  | 7.02 |  | .008 |  | .03 |  |
| Behavior ✻ Goal |  | 0.09 |  | 1 |  | 0.09 |  | 0.03 |  | .861 |  | .00 |  |
| Residual |  | 671.23 |  | 237 |  | 2.83 |  |  |  |  |  |  |  |
|  | | | | | | | | | | | | | |

**Table S6D**

*Complete Statistics for Post Hoc Acceptance ANOVA*

|  | | **Sum of Squares** | | ***df*** | | **Mean Square** | | ***F*** | | ***p*** | | **η²** | |
| --- | --- | --- | --- | --- | --- | --- | --- | --- | --- | --- | --- | --- | --- |
| Behavior |  | 31.91 |  | 1 |  | 31.91 |  | 15.14 |  | < .001 |  | .06 |  |
| Goal |  | 135.13 |  | 1 |  | 135.13 |  | 64.14 |  | < .001 |  | .21 |  |
| Behavior ✻ Goal |  | 6.09 |  | 1 |  | 6.09 |  | 2.89 |  | .090 |  | .01 |  |
| Residual |  | 499.33 |  | 237 |  | 2.11 |  |  |  |  |  |  |  |
|  | | | | | | | | | | | | | |

**Table S6E**

*Complete Statistics for Post Hoc Anxiety ANOVA*

|  | | **Sum of Squares** | | ***df*** | | **Mean Square** | | ***F*** | | ***p*** | | **η²** | |
| --- | --- | --- | --- | --- | --- | --- | --- | --- | --- | --- | --- | --- | --- |
| Behavior |  | 0.00 |  | 1 |  | 0.00 |  | 0.00 |  | .969 |  | .00 |  |
| Goal |  | 12.51 |  | 1 |  | 12.51 |  | 4.20 |  | .042 |  | .02 |  |
| Behavior ✻ Goal |  | 12.00 |  | 1 |  | 12.00 |  | 4.03 |  | .046 |  | .02 |  |
| Residual |  | 706.44 |  | 237 |  | 2.98 |  |  |  |  |  |  |  |
|  | | | | | | | | | | | | | |

**Table S6F**

*Cell Means and Standard Deviations for Specific Emotion Post Hoc ANOVAs*

| Emotion | Behavior | Outcome | *M* | *SD* |
| --- | --- | --- | --- | --- |
| Happy | Reveal | Visible | 4.25 | 0.93 |
|  |  | Invisible | 2.10 | 1.59 |
|  | Conceal | Visible | 3.29 | 1.47 |
|  |  | Invisible | 1.72 | 1.71 |
| Sad | Reveal | Visible | 2.46 | 1.59 |
|  |  | Invisible | 2.97 | 1.71 |
|  | Conceal | Visible | 3.38 | 1.94 |
|  |  | Invisible | 3.67 | 1.68 |
| Authentic | Reveal | Visible | 4.47 | 1.66 |
|  |  | Invisible | 3.94 | 1.76 |
|  | Conceal | Visible | 3.15 | 1.75 |
|  |  | Invisible | 2.53 | 1.56 |
| Accepted | Reveal | Visible | 4.07 | 1.54 |
|  |  | Invisible | 2.25 | 1.48 |
|  | Conceal | Visible | 3.04 | 1.71 |
|  |  | Invisible | 1.86 | 1.08 |
| Anxious | Reveal | Visible | 5.00 | 1.75 |
|  |  | Invisible | 5.02 | 1.79 |
|  | Conceal | Visible | 4.53 | 1.84 |
|  |  | Invisible | 5.44 | 1.52 |

**Table S7A**

*Complete Statistics for Liking ANOVA*

|  | | **Sum of Squares** | | ***df*** | | **Mean Square** | | ***F*** | | ***p*** | | **η²** | |
| --- | --- | --- | --- | --- | --- | --- | --- | --- | --- | --- | --- | --- | --- |
| Behavior |  | 28.38 |  | 1 |  | 28.38 |  | 16.30 |  | < .001 |  | .06 |  |
| Goal |  | 449.49 |  | 1 |  | 449.49 |  | 258.27 |  | < .001 |  | .52 |  |
| Behavior ✻ Goal |  | 16.06 |  | 1 |  | 16.06 |  | 9.23 |  | .003 |  | .04 |  |
| Residual |  | 412.47 |  | 237 |  | 1.74 |  |  |  |  |  |  |  |
|  | | | | | | | | | | | | | |

**Table S7B**

*Cell Means and Standard Deviations for Liking ANOVA*

| **Behavior** | **Outcome** | ***M*** | ***SD*** |
| --- | --- | --- | --- |
| Reveal | Visible | 5.12 | 1.30 |
|  | Invisible | 1.87 | 1.14 |
| Conceal | Visible | 3.95 | 1.73 |
|  | Invisible | 1.73 | 1.07 |

**Table S7C**

*Post Hoc Tukey Tests for Liking ANOVA*

| **Group 1** | **Group 2** | **Mean Difference** | **95% CI** | **Adjusted *p*** |
| --- | --- | --- | --- | --- |
| Conceal:Invisibility | Reveal:Invisibility | 0.14 | [-0.46, 0.74] | .934 |
| Conceal:Invisibility | Conceal:Visibility | 2.21 | [1.58, 2.84] | < .001 |
| Conceal:Invisibility | Reveal:Visibility | 3.38 | [2.76, 4.00] | < .001 |
| Reveal:Invisibility | Conceal:Visibility | 2.07 | [1.44, 2.70] | < .001 |
| Reveal:Invisibility | Reveal:Visibility | 3.25 | [2.63, 3.86] | < .001 |
| Conceal:Visibility | Reveal:Visibility | 1.17 | [0.53, 1.81] | < .001 |

**Table S8A**

*Complete Statistics for Ease ANOVA*

|  | | **Sum of Squares** | | ***df*** | | **Mean Square** | | ***F*** | | ***p*** | | **η²** | |
| --- | --- | --- | --- | --- | --- | --- | --- | --- | --- | --- | --- | --- | --- |
| Behavior |  | 1.57 |  | 1 |  | 1.57 |  | 0.49 |  | .485 |  | .00 |  |
| Goal |  | 45.64 |  | 1 |  | 45.64 |  | 14.23 |  | < .001 |  | .06 |  |
| Behavior ✻ Goal |  | 18.56 |  | 1 |  | 18.56 |  | 5.79 |  | .017 |  | .02 |  |
| Residual |  | 760.15 |  | 237 |  | 3.21 |  |  |  |  |  |  |  |
|  | | | | | | | | | | | | | |

**Table S8B**

*Cell Means and Standard Deviations for Ease ANOVA*

| **Behavior** | **Outcome** | ***M*** | ***SD*** |
| --- | --- | --- | --- |
| Reveal | Visible | 4.90 | 1.94 |
|  | Invisible | 5.22 | 1.85 |
| Conceal | Visible | 4.45 | 1.85 |
|  | Invisible | 5.89 | 1.50 |

**Table S8C**

*Post Hoc Tukey Tests for Ease ANOVA*

| **Group 1** | **Group 2** | **Mean Difference** | **95% CI** | **Adjusted *p*** |
| --- | --- | --- | --- | --- |
| Conceal:Invisibility | Reveal:Invisibility | -0.67 | [-1.49, 0.15] | .155 |
| Conceal:Invisibility | Conceal:Visibility | -1.44 | [-2.29, -0.58] | < .001 |
| Conceal:Invisibility | Reveal:Visibility | -0.99 | [-1.83, -0.16] | .013 |
| Reveal:Invisibility | Conceal:Visibility | -0.77 | [-1.62, 0.09] | .096 |
| Reveal:Invisibility | Reveal:Visibility | -0.32 | [-1.16, 0.52] | .750 |
| Conceal:Visibility | Reveal:Visibility | -0.44 | [-0.42, 1.31] | .550 |

**Table S9A**

*Complete Statistics for Perceived Likelihood ANOVA*

|  | | **Sum of Squares** | | ***df*** | | **Mean Square** | | ***F*** | | ***p*** | | **η²** | |
| --- | --- | --- | --- | --- | --- | --- | --- | --- | --- | --- | --- | --- | --- |
| Behavior |  | 23.23 |  | 1 |  | 23.23 |  | 6.95 |  | .009 |  | .03 |  |
| Goal |  | 35.88 |  | 1 |  | 35.88 |  | 10.74 |  | .001 |  | .04 |  |
| Behavior ✻ Goal |  | 879 |  | 1 |  | 8.79 |  | 2.63 |  | .106 |  | .01 |  |
| Residual |  | 792.02 |  | 237 |  | 3.34 |  |  |  |  |  |  |  |
|  | | | | | | | | | | | | | |

**Table S9B**

*Cell Means and Standard Deviations for Perceived Likelihood ANOVA*

| **Behavior** | **Outcome** | ***M*** | ***SD*** |
| --- | --- | --- | --- |
| Reveal | Visible | 4.76 | 2.20 |
|  | Invisible | 5.16 | 1.92 |
| Conceal | Visible | 4.96 | 1.73 |
|  | Invisible | 6.12 | 1.39 |

**Supplemental Study**

In this study, a new set of participants each rated one of four situations that we adapted from the responses gathered in Study 1. Specifically, they read vignettes describing different workplace interactions between a gay man and a coworker that correspond to different quadrants of the theoretical framework separating visibility and invisibility from revealing and concealing (Figure 1). We derived the situations depicted in these vignettes from actual experiences described by participants in Study 1. Supplemental participants then rated whether they thought the gay character in the vignette would disclose or hide his sexual orientation in that situation and how visible the gay character would be to his coworker if he disclosed and if he hid his sexual orientation.

This allowed us to examine whether participants’ ratings of what the gay character would do in each situation align with the actual behavior of participants in Study 1. We also examined whether participants’ ratings of the character’s visibility when disclosing versus hiding his sexual orientation align with the level of attention sought by Study 1 participants who faced similar situations in real life. Thus, we examined whether, even when not explicitly prompted to distinguish between concealment and invisibility, sexual minority participants would nonetheless suggest that a gay person might disclose *or* conceal their identity to either seek *or* avoid invisibility. Such a finding would provide further evidence for the utility and validity of distinguishing concealment from invisibility. We named each vignette condition for the behavior we expected the vignette to elicit (revealing or concealing) and the desirable attention outcome for that situation and behavior (visibility or invisibility), determined *a priori* based on Study 1 responses. Broadly, we hypothesized that participants would rate the vignette character as more likely to engage in the expected behavior for that condition (i.e., more likely to disclose his sexual orientation in the reveal vignettes and more likely to hide his sexual orientation in the conceal vignettes). We also anticipated that participants would judge that this expected identity management behavior would lead to the expected attention from others. Specifically, we anticipated that for the reveal/visible vignette, which involves an opportunity for affiliation with a lesbian coworker, participants would rate the gay male character as more likely to disclose his sexual orientation and would rate disclosure as leading to *more* attention than hiding (i.e., revealing his sexual orientation leads to visibility). For the reveal/invisible vignette, which involves managing unwanted romantic attention from a female coworker, we anticipated that participants would rate the gay male character as more likely to disclose his sexual orientation and would rate disclosure as leading to *less* attention than hiding (i.e., revealing his sexual orientation leads to invisibility). For the conceal/visible vignette, which involves a supervisor who will be evaluating the gay character, we anticipated that participants would rate the gay male character as more likely to hide his sexual orientation and would rate hiding as leading to *more* attention than disclosure (i.e., concealing his sexual orientation leads to visibility). Finally, for the conceal/visible vignette, which involves an interaction with a homophobic coworker, we anticipated that participants would rate the gay male character as more likely to hide his sexual orientation and would rate hiding as leading to *less* attention than disclosure (i.e., concealing his sexual orientation leads to invisibility).

In addition to participants’ ratings of the gay character’s behavior and the level of attention that would result from this behavior, we also explore participants’ ratings of how positively the character would feel when deciding to disclose or hide his sexual orientation in the situation described by the vignette.

**Method**

***Participants***

We recruited a sample of 237 lesbian, gay and bisexual participants residing in the U.S. or Canada using Prolific. *A priori* power analysis using G*Power (Faul et al., 2009) showed that this sample size offers adequate (80%) power for detection of a small-to-medium effect (Cohen’s *f* = .18) in a two-by-two between-subjects ANOVA. After excluding 11 participants who met the study’s screening criteria on Prolific but identified themselves “Straight/Heterosexual” on our own demographics survey, as well as excluding 6 participants who did not pass attention checks, we obtained a final sample of 220 participants (*M*_age_ = 30.93, *SD* = 10.04). A sensitivity analysis in G*Power (Faul et al., 2009) indicates this sample size remains sufficient to detect a small-to-medium effect (Cohen’s *f* = .19) with adequate (80%) power. This sample comprised 108 women (including 7 transgender women, 2 non-binary women, 1 non-binary transgender woman, and 1 non-binary agender woman), 92 men (including 3 transgender men, 1 non-binary man, and 2 non-binary transgender men), 13 non-binary participants, 6 participants of other genders, and 1 participant who did not indicate their gender. 127 participants reported their sexual orientation as bisexual, 69 as gay/lesbian/homosexual, 16 as pansexual, 1 as asexual, and 4 as other sexual orientations; 3 participants did not indicate their sexual orientation.

***Procedure***

**Vignettes.** Participants were randomly assigned to read one of four possible vignettes describing a workplace interaction between a gay man named Michael and his coworker, Jessica. All four vignettes describe Michael as new to the workplace and still in the process of getting to know his coworkers. During lunch, Jessica asks Michael about his plans for the weekend, presenting Michael with the choice to either tell Jessica about his plans to celebrate his anniversary with his boyfriend (i.e., to reveal his sexual orientation), or to leave out or change details to avoid talking about his relationship (i.e., to conceal his sexual orientation).

The four vignettes vary by the details they provide about Michael’s coworker, Jessica, and are adapted from the situations participants described in Study 1. We developed the vignettes to depict situations that differ in expected behavior (reveal vs. conceal), and in desirable attention outcome (visible vs. invisible). The reveal/visible vignette describes Jessica as friendly and depicts her as openly in a same-gender relationship; the reveal/invisible vignette describes Jessica as single and as having recently flirted with Michael; the conceal/visible vignette identifies Jessica as Michael’s manager and notes that she will complete his first quarterly performance evaluation the following week; and the conceal/invisible vignette notes that Michael has heard that an openly lesbian coworker left the company due to negative comments Jessica made about that coworker’s identity. The full text of the four vignettes can be found in our Supplemental Study Materials on OSF (https://osf.io/h5p4n/?view_only=bbde918f635d407ba346a38a5e79ba92).

**Ratings.** Immediately after reading the vignette, participants indicated their expectations about what Michael will do using a six-point Likert scale anchored with “Definitely not let Jessica know he is gay” and “Definitely let Jessica know he is gay”. Next, participants provided ratings about various expected outcomes in two blocks of items. Participants rated what they expected would happen if Michael decided to *disclose* his sexual orientation, and what would happen if he decided to *hide* his sexual orientation. These disclose and hide ratings were made in separate blocks presented in counterbalanced order. Participants rated how much attention they thought Michael would receive from Jessica as a result of disclosing/hiding his sexual orientation, from 1 (Much less attention) to 7 (Much more attention); and their expectations about Michael’s overall feelings and experiences of several specific feelings, which were collected as exploratory measures and so not discussed here.

**Free Response.** Participants also completed four brief free-response questions to provide insight into their construal of the situation described by the vignette. These questions included “What do you think Michael would like from this interaction with Jessica?”, “What do you think Jessica would like from this interaction with Michael?”, “What do you think will happen if Michael shares his sexual orientation?”, and “What do you think will happen if Michael hides his sexual orientation?”.

Finally, participants completed a demographics questionnaire and were debriefed and compensated.

**Results**

***Predicted Behavior***

We conducted a two-way ANOVA to determine how participants’ predictions about Michael’s disclosure decision varied according to behavior expectation condition (reveal vs. conceal) and attention expectation condition (visible vs. invisible; Figure S1). We conducted our analyses using the tidyverse (Wickham et al., 2019), sjstats (Lüdecke, 2022), and MBESS (Kelley, 2022) packages for R (Version 4.1.1; R Core Team, 2021). Consistent with our expectations, participants rated Michael as more likely to disclose his sexual orientation in the reveal vignettes (*M* = 4.83, *SD* = 1.04) than in the conceal vignettes (*M* = 3.19, *SD* = 1.46; *F*(1, 216) = 100.65, *p* < .001, η^2^ = .297, 95% CI [.219, .403]). Participants also rated disclosure as more likely in the visibility vignettes (*M* = 4.43, *SD* = 1.29) than in the invisibility vignettes (*M* = 3.65, *SD* = 1.59; *F*(1, 216) = 21.37, *p* < .001, η^2^ = .063, 95% CI [.054, .209]), although the magnitude of this effect is much smaller than the effect for behavior expectation. The interaction between expected behavior condition and expected attention condition was not significant, *F*(1, 216) = 1.67, *p* = .287, η^2^ = .003, 95% CI [.000, .050]).

**Figure S1**

*Predicted Behavior by Behavior Expectation and Attention Expectation*


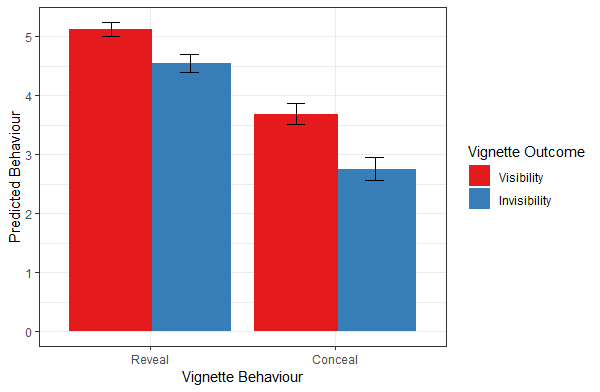


*Note.* Error bars represent standard error. Higher values on the y-axis indicate greater expectation that Michael will decide to disclose his sexual orientation.

***Attention***

To determine how vignette content affected participants’ ratings of how much attention Michael would receive from Jessica if he disclosed versus hid his sexual orientation, we conducted a three-way mixed ANOVA with expected behavior (reveal vs. conceal) and expected attention (visible vs. invisible) as between-subjects factors and with the disclosure decision rating block (disclose vs. hide) as a within-subjects factor (Figure S2; Tables S10A & S10B). We conducted this analysis using jamovi (The jamovi project, 2023) to facilitate post-hoc Tukey testing for mixed ANOVA. We found that the three-way interaction was significant: As expected, participants’ beliefs about the attention Michael would receive were sensitive to the situation depicted in the vignette. That said, these within-participant differences diverged somewhat from our expectations. We expected that participants would rate that Michael receives the desirable attention level (visibility or invisibility) for a given vignette condition if he performs the expected behavior (reveal or conceal) for a that vignette (e.g., in the reveal/invisible condition, participants would rate Michael as less visible if he reveals his sexual orientation than if he conceals). Consistent with these expectations, post hoc Tukey tests showed that participants rated that Michael would receive more attention when disclosing his sexual orientation than when hiding his sexual orientation in the reveal/visible condition (i.e., they believed revealing would lead to visibility), *t*(216) = 3.72, *p* = .006, and in the conceal/invisible condition (i.e., they believed concealing would lead to invisibility), *t*(216) = 4.32, *p* < .001. Participants likewise rated that Michael would receive less attention when disclosing his sexual orientation in the reveal/invisible condition (i.e., they believed revealing would lead to invisibility), *t*(216) = -8.52, *p* < .001. However, whereas we expected that participants would rate that Michael would receive more attention when hiding than when disclosing in the conceal/visible condition (i.e., that concealing would lead to visibility), we instead found a marginal trend whereby participants expected Michael to receive *more* attention when disclosing in this condition (i.e., they instead believed that concealing would lead to invisibility and not visibility), *t*(216) = 3.02, *p* = .055.

**Table S10A**

*ANOVA Outputs for Effects of Behavior Expectation, Desirable Outcome, and Disclosure Decision on Attention Ratings: Within-Subjects Effects*

|  | |  |  | |  |  |  |  |  |  |  |  |  |  |  |
| --- | --- | --- | --- | --- | --- | --- | --- | --- | --- | --- | --- | --- | --- | --- | --- |
|  | | | **Sum of Squares** | | | **df** | | **Mean Square** | | **F** | | **p** | | **η²** | |
| Disclosure Decision | |  | 4.41 | |  | 1 |  | 4.41 |  | 1.86 |  | .17 |  | .00 |  |
| Disclosure Decision ✻ Expected Behavior | |  | 85.61 | |  | 1 |  | 85.61 |  | 36.15 |  | < .001 |  | .06 |  |
| Disclosure Decision ✻ Desirable Outcome | |  | 71.27 | |  | 1 |  | 71.27 |  | 30.10 |  | < .001 |  | .05 |  |
| Disclosure Decision ✻ Expected Behavior ✻ Desirable Outcome | |  | 102.81 | |  | 1 |  | 102.81 |  | 43.42 |  | < .001 |  | .07 |  |
| Residual | |  | 511.47 | |  | 216 |  | 2.37 |  |  |  |  |  |  |  |
|  | | | | | | | | | | | | | | | |
| **Table S10B**  *ANOVA Outputs for Effects of Behavior Expectation, Desirable Outcome, and Disclosure Decision on Attention Ratings: Between-Subjects Effects* | | | | | | | | | | | | | | | |
|  | | | | | | | | | | | | | | | |
|  |  |  |  |  |  |  | |  |  |  |  |  |  |  |  |
|  | | **Sum of Squares** | | **df** | | **Mean Square** | | | **F** | | **p** | | **η²** | |  |
| Expected Behavior |  | 6.42 |  | 1 |  | 6.42 | |  | 6.48 |  | .01 |  | .00 |  |  |
| Desirable Outcome |  | 22.31 |  | 1 |  | 22.31 | |  | 22.51 |  | < .001 |  | .02 |  |  |
| Behavior Expectation ✻ Desirable Outcome |  | 3.57 |  | 1 |  | 3.57 | |  | 3.61 |  | .06 |  | .00 |  |  |
| Residual |  | 214.12 |  | 216 |  | 0.99 | |  |  |  |  |  |  |  |  |
| *Note*. Type 3 Sums of Squares | | | | | | | | | | | | | | |  |

**Figure S2**

*Predicted Attention Level by Behavior Expectation, Desirable Outcome, and Disclosure Decision*

**
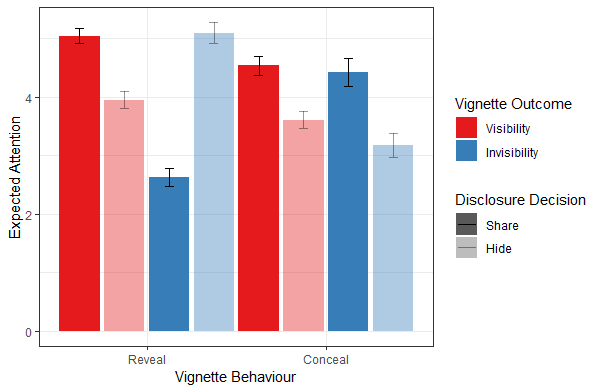
**

*Note.* Error bars represent standard error.

***Exploratory Analysis: Variation by Sexual Orientation***

To explore whether different minority sexual orientations give rise to different appraisals of the situations in the vignettes, we conducted an exploratory analysis with participant sexual orientation as a quasi-independent variable. Specifically, we grouped participants according to whether they experience exclusive same-gender attraction (i.e., participants who identified themselves as gay/lesbian/homosexual; *n* = 69) or experience attraction to multiple genders (i.e., participants who identified themselves as bisexual or as pansexual; *n* = 143). We excluded 8 participants who reported other sexual orientations or did not indicate their sexual orientation from analysis.

We then conducted a three-way ANOVA examining the effects of behavior expectation condition (reveal vs. conceal), attention expectation condition (visible vs. invisible), and participant sexual orientation (gay/lesbian vs. bisexual/pansexual) on participants’ predictions about Michael’s behavior. Due to the unequal cell sizes in this analysis, we performed Levene’s test to verify whether the assumption of homogeneity of variance was met. This yielded a marginally significant result (*F*(7, 204) = 2.04, *p* = .052), so we applied White’s heteroskedasticity correction. We found no main effect of participant sexual orientation (*F*(1, 204) = 0.08, *p* = .783). All effects involving participant sexual orientation were likewise not significant (all *F*s(1, 204) ≤ 0.23, all *p*s ≥ .631), indicating that gay/lesbian and bisexual/pansexual participants made similar predictions about Michael’s behavior.

We refrain from conducting a comparable exploratory analysis on the effects of participant sexual orientation (gay/lesbian vs. bisexual/pansexual), expected behavior (reveal vs. conceal), expected attention (visible vs. invisible), and rating block (disclose vs. hide) on the amount of attention participants expect Michael to receive, on the basis that an unbalanced sample of *N* = 202 does not yield sufficient statistical power to draw meaningful conclusions from a four-way analysis. At the pattern level, response tendencies appear similar across gay/lesbian and bisexual/pansexual participants (Figures S3A and S3B), but we advise due caution in the interpretation of this result in the absence of inferential statistical support.

**Figure S3A**

*Gay and Lesbian Participants’ Ratings of Predicted Attention Level by Behavior Expectation, Desirable Outcome, and Disclosure Decision*

*
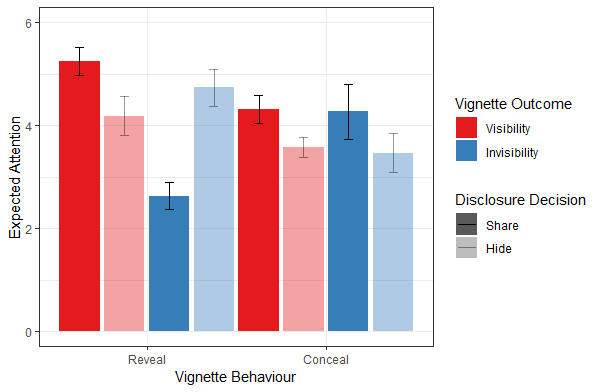
*

*Note.* Error bars represent standard error.

**Figure S3B**

*Bisexual and Pansexual Participants’ Ratings of Predicted Attention Level by Behavior Expectation, Desirable Outcome, and Disclosure Decision*

*
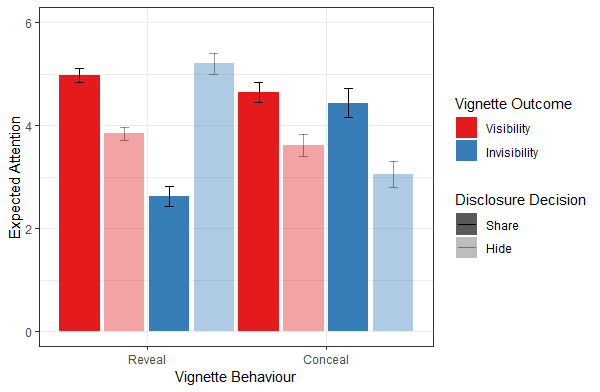
*

*Note.* Error bars represent standard error.

**Discussion**

In this study, we found that participants’ predictions about the behavior of a hypothetical gay man were consistent with the reported behavior of the Study 1 participants who had faced such situations in their real lives. That is, participants thought that Michael would reveal his sexual orientation in the vignettes adapted from situations where Study 1 participants had described revealing their sexual orientation, and thought that Michael would conceal his sexual orientation in the vignettes adapted from situations where Study 1 participants had described concealing their sexual orientation. We also found partial support for the distinction between invisibility and concealment: Participants who read the vignette situation that we expected would elicit revealing sexual orientation to become invisible did correspondingly predict that Michael would receive less attention if he decided to disclose his sexual orientation, demonstrating an understanding that revealing one’s sexual orientation can promote invisibility (rather than visibility) under certain circumstances.

On the other hand, we did not find this distinction amongst participants assigned to the vignette meant to elicit concealing one’s sexual orientation to remain visible: These participants predicted that Michael would receive marginally more attention when revealing his sexual orientation. It is possible that in the conceal/visible vignette (in which Michael interacts with a supervisor who will be reviewing his performance), participants expected that disclosure would lead to immediate social attention (even if it could interfere with the evaluation of Michael’s work). Indeed, these participants’ free responses (which describe what they believe the characters want and what they believe will happen if Michael discloses or hides his sexual orientation) rarely expressed concerns about the performance review. Instead, most participants’ responses described disclosure as a way for Michael to grow closer with his supervisor. Thus, many participants in this condition may have construed this situation as an opportunity to reveal to become socially visible for the sake of affiliation, similar to the reveal/visible vignette, rather than anticipating that revealing could make the character’s contributions at work less visible, although some participants did express concerns about how Michael will be evaluated.^[[1]](#footnote-1)^ It may be that certain participants are dispositionally or situationally less attuned to, for instance the potential adverse consequences of revealing one’s sexual orientation to a workplace superior. Thus, it might follow that the patterns of experience we observed under Study 1’s nonrandom qualitative analysis (in which participants were grouped according to the responses they generated, which may reflect underlying personal tendencies) do not emerge when true random assignment is enforced and participants who may be less sensitive to potential negative effects of disclosure remain assigned to concealment conditions.

Moreover, in addition to participants’ ratings of Michael’s behavior strongly differing by expected behavior condition, these predictions also differed by desirable attention condition. Participants rated that Michael would be more likely to reveal his sexual orientation in the visible vignette conditions than in the invisible vignette conditions (though this effect was not as strong as the effect of expected disclosure behavior). Thus, we see that although participants show evidence of meaningfully distinguishing between concealment and invisibility, this distinction is not perfect and some overlap between the two emerges when predicting behavior across these specific situations.

These findings are notably limited by the number of distinct situations presented in each vignette condition. Although we originally conceived of this study as a confirmatory experiment that adapted multiple situations from Study 1 for each of the four vignette conditions, we were constrained by the lack of diversity in Study 1 responses that described revealing to become invisible, such that the Supplemental Study only presented one vignette for each condition. Thus, whether these effects would generalize across a broader range of stimuli remains to be determined. In a similar vein, it may be that attributes of the vignette content (e.g., their wording) or of the participants themselves influenced participants’ construal of the vignettes in unanticipated ways. For instance, the implied risk of Michael receiving a poor performance evaluation after disclosure in the conceal/visible condition may not have been salient to some or all participants depending on how they interpreted the text and their own dispositional sensitivity to the risks associated with disclosing a minority sexual identity in the workplace.

We furthermore note that, whereas we assess predicted attention as a dependent variable in this work, our measure is blind to the nature of this attention. For instance, although we would anticipate that the attention participants predict Michael would receive when coming out to an openly gay coworker is qualitatively different from the attention they predict he would receive when coming out to a homophobic coworker, our attention measure does not capture this distinction. We use attention as a proxy for visibility—specifically, whether one is deemed relevant or irrelevant—but lose some resolution in our capacity to identify stigmatizing, threat-based relevance. This complicates our interpretation of certain findings pertaining to attention. In particular, our attention measure offers limited insight into participants’ reasoning for the unanticipated pattern of attention predictions for the conceal/visible vignette, leading us to refer to participants’ free responses to better understand their construal of the situation. All the same, we see that participants’ predictions concerning Michael’s behavior and the attention he would receive are consistent with our framework for the three other vignettes.

**References**

Faul, F., Erdfelder, E., Buchner, A., & Lang, A.-G. (2009). Statistical power analyses using G*Power 3.1: Tests for correlation and regression analyses. Behavior Research Methods, 41(4), 1149–1160. <http://doi.org/10.3758/BRM.41.4.1149>

Kassambara, A. (2022). rstatix: Pipe-friendly framework for basic statistical tests (Version 0.7.1) [R package]. <https://CRAN.R-project.org/package=rstatix>

Kelley, K. (2022). MBESS: The MBESS R package (Version 4.9.2) [R package]. <https://CRAN.R-project.org/package=MBESS>

Kenrick, D. T., Neuberg, S. L., Griskevicius, V., Becker, D. V., & Schaller, M. (2010). Goal-driven cognition and functional behavior: The fundamental-motives framework. *Current Directions in Psychological Science*, *19*(1), 63–67. https://doi.org/[10.1177/0963721409359281](https://doi-org.myaccess.library.utoronto.ca/10.1177/0963721409359281)

Krems, J. A., Kenrick, D. T., & Neel, R. (2017). Individual perceptions of self-actualization: What functional motives are linked to fulfilling one’s full potential? *Personality and Social Psychology Bulletin, 43*(9), 1337–1352. <https://doi.org/10.1177/0146167217713191>

Lüdecke, D. (2022). sjstats: Statistical functions for regression models (Version 0.18.2) [R package]. <http://doi.org/10.5281/zenodo.1284472>

Navarro, D. (2015). Learning statistics with R: A tutorial for psychology students and other beginners (Version 0.5.1) [R package]. <https://learningstatisticswithr.com>

Neel, R., Kenrick, D., White, A. E., & Neuberg, S. (2016). Individual differences in fundamental social motives. *Journal of Personality and Social Psychology*, *110*(6), 887-907. <https://doi.org/10.1037/pspp0000068>

Pirlott, A. G., & Neuberg, S. L. (2014). Sexual prejudice: Avoiding unwanted sexual interest? *Social Psychological and Personality Science, 5*(1), 92–101. <https://doi.org/10.1177/1948550613486674>

R Core Team (2021). R: A language and environment for statistical computing. R Foundation for Statistical Computing, Vienna, Austria. https://www.R-project.org/

Schaller, M., Kenrick, D., Neel, R., & Neuberg, S. (2017). Evolution and human motivation: A fundamental motives framework. *Social and Personality Psychology Compass*, *11*(6), Article e12319. <https://doi.org/10.1111/spc3.12319>

The jamovi project (2023). jamovi (Version 2.3) [Computer Software]. Retrieved from <https://www.jamovi.org>

Wickham, H., Averick, M., Bryan, J., Chang, W., McGowan, L. D., François, R., Grolemund, G., Hayes, A., Henry, L., Hester, J., Kuhn, M., Pedersen, T. L., Miller, E., Bache, S. M., Müller, K., Ooms, J., Robinson, D., Seidel, D. P., Spinu, … & Yutani, H. (2019). Welcome to the tidyverse. Journal of Open Source Software, 4(43), 1686. <http://doi.org/10.21105/joss.01686>

1. We note that difference scores for participants’ attention predictions (i.e., their belief that the character would receive either increased or decreased attention when disclosing vs. concealing his sexual orientation) in the conceal/visible condition were not associated with participants’ age (Pearson’s *r* = -.12, *p* = .39), indicating that participant age does not account for these varying interpretations of the vignette. [↑](#footnote-ref-1)
